# A single-cell map of dynamic chromatin landscapes of immune cells in renal cell carcinoma

**DOI:** 10.1038/s43018-022-00391-0

**Published:** 2022-06-06

**Authors:** Nikos Kourtis, Qingqing Wang, Bei Wang, Erin Oswald, Christina Adler, Samvitha Cherravuru, Evangelia Malahias, Lance Zhang, Jacquelynn Golubov, Qiaozhi Wei, Samantha Lemus, Min Ni, Yueming Ding, Yi Wei, Gurinder S. Atwal, Gavin Thurston, Lynn E. Macdonald, Andrew J. Murphy, Ankur Dhanik, Matthew A. Sleeman, Scott S. Tykodi, Dimitris Skokos

**Affiliations:** 1 Regeneron Pharmaceuticals, Tarrytown, NY USA; 2 Department of Medicine, Division of Medical Oncology, University of Washington and Clinical Research Division, Fred Hutchinson Cancer Research Center, Seattle, WA USA

**Keywords:** Renal cancer, Tumour immunology, Cancer, Cancer genomics

## Abstract

A complete chart of the chromatin regulatory elements of immune cells in patients with cancer and their dynamic behavior is necessary to understand the developmental fates and guide therapeutic strategies. Here, we map the single-cell chromatin landscape of immune cells from blood, normal tumor-adjacent kidney tissue and malignant tissue from patients with early-stage clear cell renal cell carcinoma (ccRCC). We catalog the T cell states dictated by tissue-specific and developmental-stage-specific chromatin accessibility patterns, infer key chromatin regulators and observe rewiring of regulatory networks in the progression to dysfunction in CD8^+^ T cells. Unexpectedly, among the transcription factors orchestrating the path to dysfunction, NF-κB is associated with a pro-apoptotic program in late stages of dysfunction in tumor-infiltrating CD8^+^ T cells. Importantly, this epigenomic profiling stratified ccRCC patients based on a NF-κB-driven pro-apoptotic signature. This study provides a rich resource for understanding the functional states and regulatory dynamics of immune cells in ccRCC.

## Abstract (web summary)

Skokos and colleagues characterize human early-stage clear cell renal cell carcinoma with single-cell ATAC-seq and RNA-seq, identifying a spectrum of NFκB-promoted T cell dysfunction in the microenvironment and defining a pro-apoptotic signature.

## Main

The tumor microenvironment is a complex ecosystem composed of heterogeneous cell types. The composition and functional state of tumor-infiltrating immune cells have critical roles in tumor development^1^. Immunotherapies have revolutionized cancer treatment, resulting in sustained clinical responses when treating tumors of diverse origins^2^. Nevertheless, the efficacy of immunotherapy is not uniform across cancer types, and the majority of patients still succumb to disease. Therefore, it is imperative to uncover the mechanisms that drive or hinder effective responses to immunotherapy.

Key factors associated with clinical outcome include the number and functional state of T cells infiltrating the tumor at baseline and during treatment^3^. Recent single-cell-based transcriptomic analyses of tumor-infiltrating lymphocytes reveal extensive heterogeneity, which may influence therapeutic outcome^4–6^. In addition to naive, effector, memory and regulatory T cells, a substantially more heterogeneous T cell compartment that displays features of dysfunction is frequently observed^4,7^. Dysfunctional T cells are characterized by impaired production of cytokines and cytotoxic molecules and by increased surface expression of inhibitory receptors^8^. Intriguingly, dysfunctional T cells in models of chronic viral infection and in murine and human cancers harbor unique chromatin accessibility patterns^9–11^. Moreover, current immunotherapies cannot epigenetically reprogram these dysfunctional T cells; therefore, durable responses are impeded^10^. Recent findings have started to elucidate the transcriptional networks that mediate T cell dysfunction^9,11–13^. At present, the nature of the regulatory circuit that orchestrates T cell transition along the naive-to-dysfunction path in cancer is unclear.

Renal cell carcinoma (RCC) is known to be responsive to immune-based therapies, and the development of immune-checkpoint inhibitors has transformed the management of advanced-stage RCC^14–18^. Nonetheless, the majority of patients either have primary resistance to therapies or develop resistance after an initial response^16–19^. RCC displays unique characteristics compared with other immune-responsive solid tumors, including a modest mutation burden^20^ and association of increased infiltration of CD8^+^ T cells with worse prognosis^21^. The latter paradox can be explained, in part, by high heterogeneity in the activation and cytotoxic potential of infiltrating T cells^22–26^. Therefore, to develop novel and improved immune-based treatments in RCC, an understanding of the developmental and functional states of immune cells in patients is of paramount importance.

Here, we generate an epigenetic map of the evolution of immune cell states and examine in detail the regulatory landscape of T cells in patients with ccRCC. To gain insight into the epigenetic regulation of lymphocytes in RCC, we employ single-cell assay technology for transposase-accessible chromatin using sequencing (scATAC-seq)^27^. Through surveying the chromatin landscapes of T cells of malignant and nonmalignant tissues from patients with ccRCC, we observe cell-type-specific and tissue-specific chromatin accessibility patterns. Furthermore, analysis of intratumoral CD8^+^ T cells demonstrates a continuum of dysfunctional states and an extensive remodeling of the accessibility of regulatory elements. Paradoxically, we observed enrichment of the NF-κB-binding motif in the late dysfunctional CD8^+^ T cell subset. Our data provide a valuable resource for dissecting the epigenetic and transcriptional heterogeneity of T cells in ccRCC and have the potential to guide therapeutic strategies based on a patient’s immune cell fate repertoire.

## Results

### Single-cell chromatin landscapes of immune cells in ccRCC

**Fig. 1:**
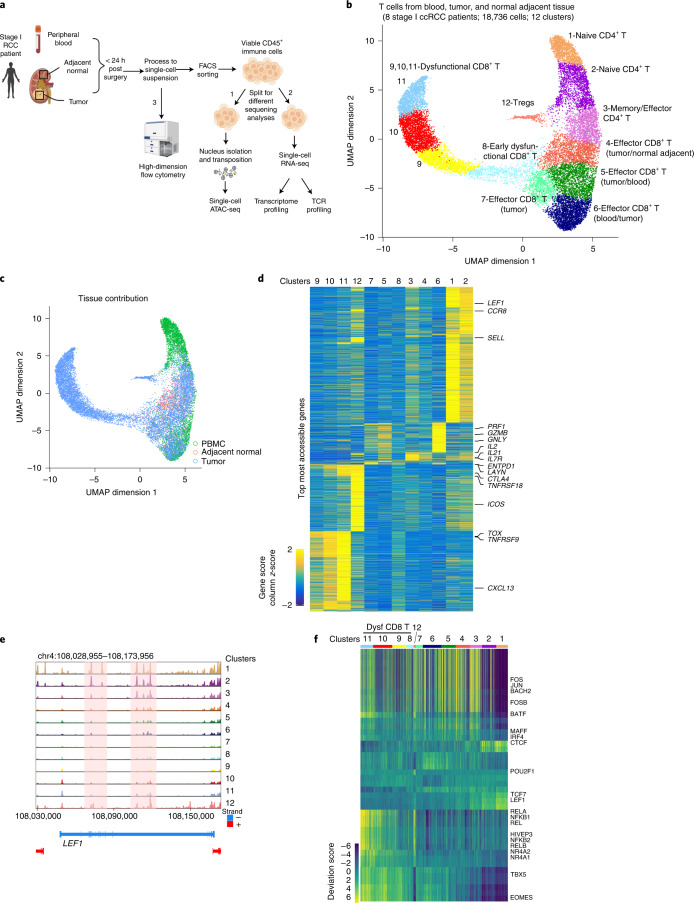
Single-cell chromatin accessibility of T cells in ccRCC. **a**, Schematic of chromatin accessibility, transcriptome and TCR profiling, and validation at the protein level of cells from peripheral blood, tumor tissue and adjacent normal tissue in early-stage ccRCC patients. Imaged created with BioRender.com. **b**, UMAP projection of 18,736 scATAC-seq profiles of T cells from peripheral blood, tumor tissue and adjacent normal tissue combining eight patient samples. Dots represent individual cells and colors indicate cluster identity, specified next to each cluster. **c**, UMAP projection of T cells colored by tissue of origin. **d**, Heatmap of gene activity scores of the most accessible genes in each cluster (LFC ≥ 1, FDR ≤ 0.05 compared with other clusters) derived from **b**. **e**, Genome tracks of aggregate scATAC-seq data visualization of the *LEF1* locus, clustered as indicated in **b**. **f**, Heatmap representation of ATAC-seq chromVAR bias-corrected deviations in the 49 most variable TFs across all scATAC-seq clusters. Cluster identities are indicated at the top of the plot.

**Extended Data Fig. 1:**
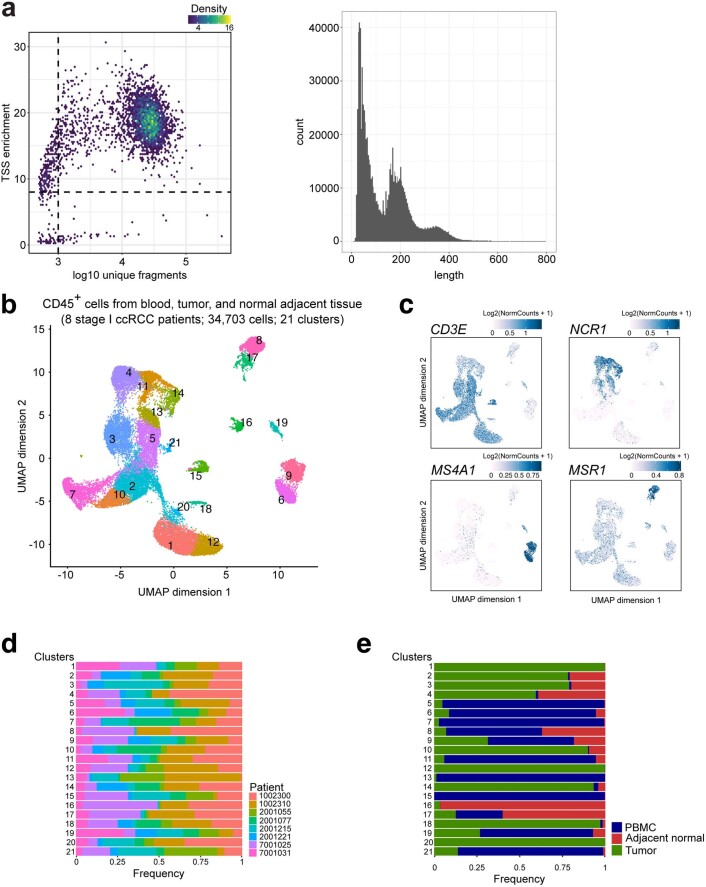
Single-cell chromatin accessibility of immune cells in ccRCC. **a**, Representative scATAC-seq data quality control filters of a patient sample (patient 1002300, tumor; left). The x-axis shows the number of unique ATAC-seq nuclear fragments (in log scale) in each single cell (each dot) and the y-axis shows the transcriptional start sites (TSS) enrichment of all fragments in that cell. Dashed lines represent the filters for high-quality single-cell data (at least 1,000 unique fragments and TSS score great than or equal to 8). Representative plot of scATAC-seq fragment size distributions demonstrating sub-, mono-, and multi-nucleosome spanning ATAC-seq fragments (right). **b**, UMAP projection of 34,703 scATAC-seq profiles of CD45^+^ cells from peripheral blood, tumor, and adjacent normal tissue combining eight patient samples. Dots represent individual cells and colors indicate cluster identity. **c**, UMAP projection of immune cells colored by gene scores, reflecting the general chromatin accessibility of the indicated gene. **d**, Patient-specific contribution of cells in each cluster derived from **b**. **e**, Tissue-specific contribution of cells in each cluster derived from **b**. Source data

To catalog the heterogeneity of epigenetic states of immune cells within ccRCC patients, we generated scATAC-seq profiles (10x Genomics platform)^27^ of immune cells (CD45^+^) isolated from blood and from malignant and normal adjacent kidney tissues of eight patients with early-stage ccRCC (Fig. 1a). In total, we generated scATAC-seq profiles of 34,703 immune cells. To exclude low-quality cells, we filtered scATAC-seq data using cut-offs of 1,000 unique nuclear fragments per cell and a transcription start site (TSS) enrichment score of 8, as previously described^27^ (Extended Data Fig. 1a; Methods). These scATAC-seq profiles also exhibited fragment-size periodicity and high enrichment of fragments at TSSs (Extended Data Fig. 1a; Methods). Next, a graph-based clustering of the immune cells was performed based on their chromatin accessibility landscape, resulting in a total of 21 major clusters (Extended Data Fig. 1b). To classify these clusters, we computed the gene activity scores of known immune-lineage-defining marker genes in each cluster, thereby evaluating the general accessibility of the gene (Methods)^27–29^. Annotation of cell types using this method led to identification of all expected cell types (Extended Data Fig. 1c). Importantly, the distribution of immune cells from different patients across the clusters suggested no patient-specific cellular epigenetic states or batch effects (Extended Data Fig. 1b, d). Moreover, we observed clusters dominated by cells from specific tissues, suggesting that tissue residency affects the epigenetic landscape of some immune cells (Extended Data Fig. 1e). Thus, the single-cell epigenetic approach revealed a complex composition of immune cell fates in ccRCC.

### T cells form a continuum of epigenetic states in ccRCC

**Extended Data Fig. 2:**
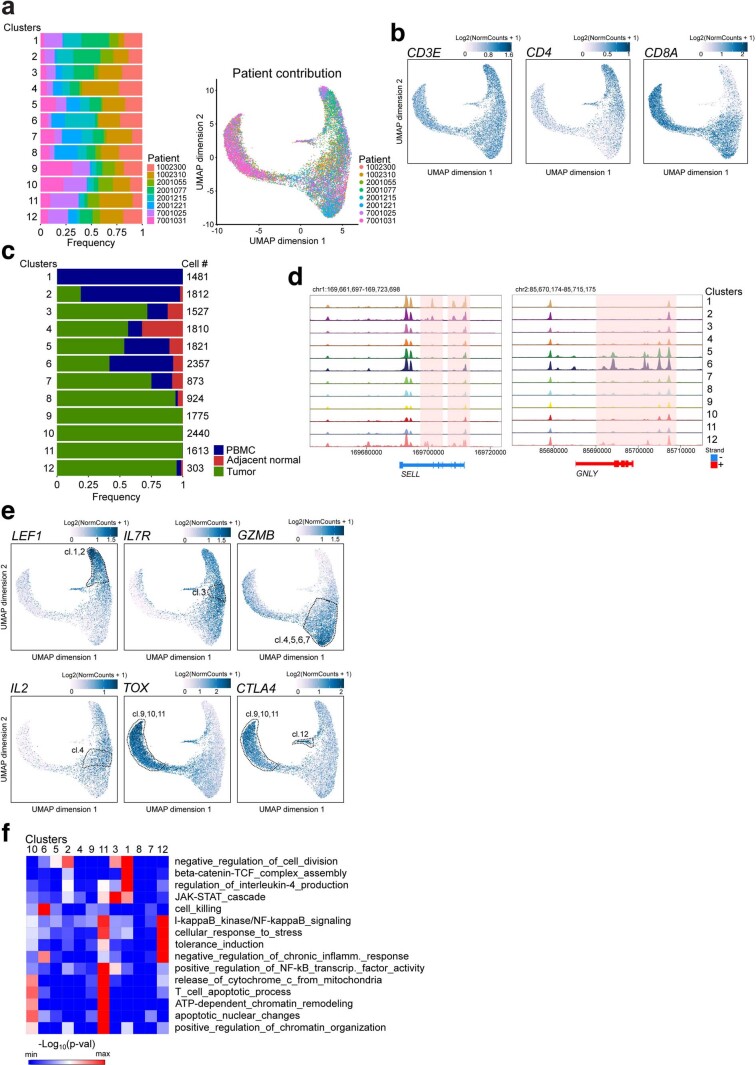
*Cis*-regulatory elements in open chromatin regions of T cells in ccRCC. **a**, Patient-specific contribution of T cells in each cluster derived from Fig. 1b (left), and UMAP projection (right). **b**, UMAP projection of T cells colored by gene activity scores of the indicated gene (n = 18736 cells). **c**, Tissue-specific contribution of T cells in each cluster. **d**, Genome tracks of aggregate scATAC-seq data visualization of the *SELL* and *GNLY* loci, clustered as indicated in Fig. 1b. **e**, UMAP projection of T cells colored by gene activity scores of the indicated gene. **f**, Enrichment of Gene Ontology terms associated with the top accessible cis-regulatory elements of at least one cluster (LFC > 1.5, FDR < 0.01 compared to other clusters), across all clusters. Selected Gene Ontology terms that are significantly enriched (adjusted binomial p-value or adjusted hypergeometric p-value<0.05; here term enrichment was calculated using either the binomial test or the hypergeometric test, with p-values corrected with the Benjamini-Hochberg procedure) in at least one cluster are shown. Analysis was done using the software tool GREAT. Here, Benjamini-Hochberg corrected binomial p-values are shown. Source data

T cells are the key target population for cancer immunotherapies and the identification of effective biomarkers requires deep understanding of T cell states. Therefore, we identified a population of immune cells with high gene activity scores for known T cell markers (*CD3D*, *CD8A* and *CD4*) and re-clustered this subset of 18,736 T cells based on the chromatin accessibility landscape. We identified 12 T cell subclusters with a rich diversity of chromatin landscapes and cell states (Fig. 1b). T cell chromatin accessibility clustering did not exhibit patient-specific effects (Extended Data Fig. 2a), consistent with previous immune single-cell transcriptomic studies^7^. Examination of gene accessibility in *CD4* and *CD8* loci identified four CD4^+^ and eight CD8^+^ T cell clusters (Extended Data Fig. 2b). These clusters exhibited distinct tissue distribution for T cells. For example, cluster C1_CD4 contained mainly cells from blood, whereas clusters C8,9,10,11_CD8 and C12_CD4 were almost exclusively populated with cells from tumor tissue (Fig. 1c and Extended Data Fig. 2c). To classify each cluster, we examined the most accessible (log-fold change (LFC) ≥ 1, false discovery rate (FDR) ≤ 0.05) genes in each cluster (Fig. 1d). Cells of C1,2_CD4 type, which were predominant in peripheral blood, demonstrated distinctly high chromatin accessibility for naive marker genes including *LEF1* and *SELL* (Fig. 1d,e and Extended Data Fig. 2d,e). The C3_CD4 cluster, composed of CD4^+^ T cells mainly from tumor tissue, had high accessibility for *IL7R* and *IL2*, consistent with a memory- and/or effector-like fate^4^ (Fig. 1d and Extended Data Fig. 2e). In addition, cells in this cluster displayed increased accessibility for T_FH_-specific genes such as *IL21*, a cell population recently implicated in antitumor immunity^30,31^ (Fig. 1d). C5,6,7_CD8, which was predominantly composed of CD8^+^ T cells from tumor tissue and blood, exhibited high gene accessibility of cytotoxic molecules including *GNLY*, *PRF1* and *GZMB*, with cells in C6_CD8 exhibiting the highest accessibility for these cytolytic molecules (Fig. 1d and Extended Data Fig. 2d,e). C4_CD8 was mainly composed of CD8^+^ T cells from malignant and adjacent normal kidney tissue (Fig. 1c and Extended Data Fig. 2c). Compared with those in C5,6_CD8, the C4_CD8 T cells had markedly reduced gene accessibility of effector molecules such as *GNLY* (Fig. 1d and Extended Data Fig. 2d). However, C4_CD8 was characterized by high gene accessibility for *IL2*, suggesting that some level of effector function was retained in these cells (Fig. 1d and Extended Data Fig. 2e).

C9,10,11_CD8 cells were almost exclusively derived from tumor tissue and were characterized by a distinct chromatin landscape that clustered separately (Fig. 1b, c and Extended Data Fig. 2c). These cells displayed high gene accessibility for multiple dysfunction-related genes including *TOX*, *LAYN*, *ENTPD1*, *CTLA4* and *CXCL13* (refs. ^7,13,32^) (Fig. 1d and Extended Data Fig. 2e). For C8_CD8, which was mainly composed of CD8^+^ T cells from tumors (Fig. 1c and Extended Data Fig. 2c), we observed a pattern of gene accessibility that resembled that of C9,10,11_CD8, albeit at a lower intensity, suggesting that cells in this cluster had an early dysfunction fate (Fig. 1d). C12_CD4, which mainly comprised CD4^+^ T cells from tumors (Fig. 1c and Extended Data Fig. 2c), was associated with high gene accessibility for markers of tumor-infiltrating regulatory T cells (Tregs), including *TNFRSF18*, *ICOS* and *CTLA4* (Fig. 1d and Extended Data Fig. 2e). Given the strong correlation between the functional status of tumor-infiltrating T cells and patient prognosis^21^, we investigated the epigenetic landscape of these cells. To further examine the identities of T cell clusters, we applied GREAT gene ontology enrichment analysis^33^ to the highly accessible potential regulatory elements in each cluster (FDR < 0.05; LFC > 1) to evaluate putative biological processes associated with these regulatory elements. This unbiased annotation demonstrated the enrichment of gene ontology terms for biological processes related to naive, effector/memory and regulatory fates (Extended Data Fig. 2f). Combined, our data reveal a diverse regulatory landscape of T cells in ccRCC patients.

### A catalog of transcription factor programs in ccRCC

**Extended Data Fig. 3:**
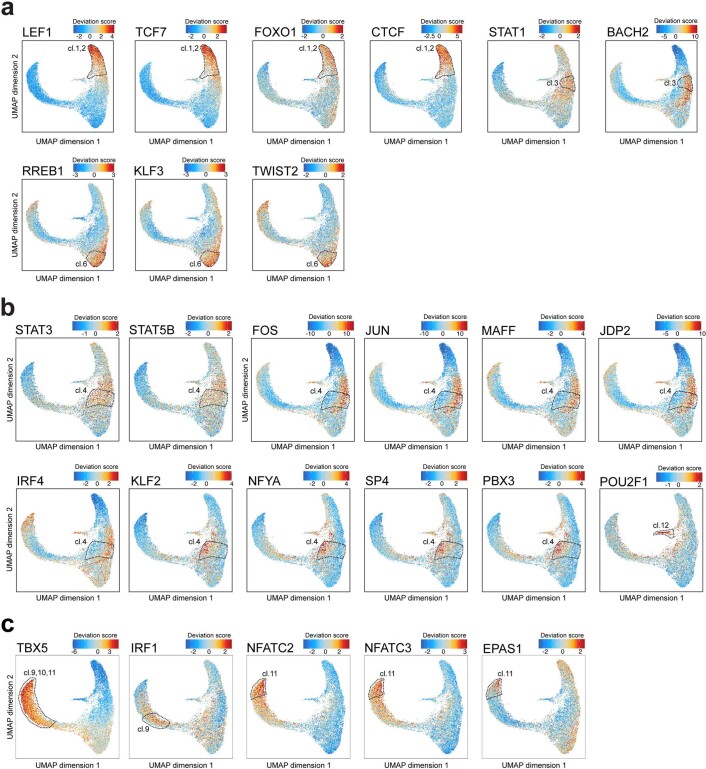
TF motif enrichment in open chromatin regions of T cells in ccRCC. **a**, UMAP projection of T cell scATAC-seq profiles colored by chromVAR TF motif bias-corrected deviations for the indicated TFs (two-sided Wilcoxon rank sum test; p < 2.2×10^−16^ for the TFs in the indicated clusters -CD4 clusters: LEF1, TCF7, FOXO1, TCF7, STAT1, BACH2 and CD8 clusters: RREB1, KLF3, TWIST2- compared to the other CD4 or CD8 T cell clusters, respectively). **b**, UMAP projection of T cell scATAC-seq profiles colored by chromVAR TF motif bias-corrected deviations for the indicated TFs (two-sided Wilcoxon rank sum test; p < 2.2×10^−16^ for STAT3 and STAT5 in C4_CD8 compared to the other CD8 T cell clusters, p < 2.2×10^−16^ for POU2F1 in C12_CD4 compared to the other CD4 T cell clusters). **c**, UMAP projection of T cell scATAC-seq profiles colored by chromVAR TF motif bias-corrected deviations for the indicated TFs (two-sided Wilcoxon rank sum test; for TBX5, p < 2.2×10^−16^ for C11_CD8 compared to C10_CD8 or C9_CD8, p < 2.2×10^−16^ for C9,10,11_CD8 compared to the other clusters; for IRF1, NFATC2, NFATC3, and EPAS1, p < 2.2×10^−16^ for the indicated cluster compared to the other clusters; **a**, **b**, **c**, n = 18736 cells).

Having mapped the epigenetic landscape of T cells across blood and normal and malignant kidney tissue, we then sought to identify dynamic TF regulatory programs in various T cell fates. The ATAC-seq method enables the inference of TF activity^27,34,35^. By analyzing TF activity, we identified cell-type-specific, fate-specific and tissue-specific binding programs (Fig. 1f). Specifically, for CD4^+^ T cells, the C1,2_CD4 clusters were characterized by high TF activity for “naive” TFs including LEF1, TCF7 and FOXO1 (Fig. 1f and Extended Data Fig. 3a). Notably, in these clusters we observed motif enrichment for CTCF, an architectural factor that organizes higher-order chromatin structure^36^ (Fig. 1f and Extended Data Fig. 3a). C3_CD4 exhibited motif enrichment for TFs related to immune activation such as STAT1 (Extended Data Fig. 3a) and early-differentiated memory T cells such as BACH2 (Extended Data Fig. 3a). C6_CD8, which was composed of CD8^+^ T cells with high effector function and a substantial contribution from the blood (Fig. 1c,d and Extended Data Fig. 2c), was enriched for motifs of less-described TFs including RREB1, KLF3 and the bHLH family TF TWIST2 (Extended Data Fig. 3a), in addition to TFs associated with effector function such as EOMES.

### Heterogeneity of chromatin states of T cell dysfunction

**Fig. 2:**
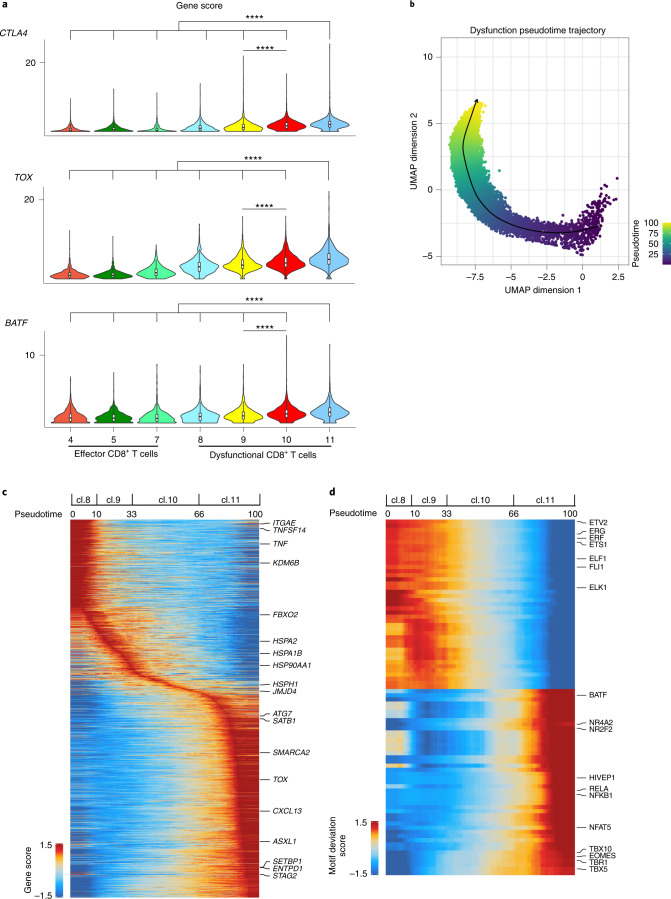
The chromatin landscape of dysfunctional T cells. **a**, Violin plots of gene activity scores of the indicated genes for effector/nondysfunctional (C4,5,7_CD8) and dysfunctional (C8,9,10,11_CD8) T cell clusters. Pairwise comparisons of gene activity scores for the indicated gene between specified T cell clusters were determined using a two-sided Wilcoxon rank-sum test. The *P* values obtained were subjected to multi-test correction with the FDR method (****adjusted *P* < 0.0001; raw adjusted *P* values are listed in the source data; for clusters 4, 5, 7, 8, 9, 10, 11, *n* = 1,810, 1,821, 873, 924, 1,775, 2,440 and 1,613 cells, respectively). **b**, Cell alignment to the pseudotime developmental trajectory within the dysfunctional CD8^+^ T cell populations. The smoothened arrow represents a visualization of the interpreted trajectory in the UMAP embedding. **c**, Pseudotime heatmap ordering of gene activity scores of the top 10% most variable genes across the potential CD8^+^ T cell dysfunction trajectory. General ordering of cells from different dysfunction clusters along the pseudotime course is marked along the trajectory at the top. **d**, Pseudotime heatmap ordering of the top 10% most variable chromVAR TF motif bias-corrected deviations in the CD8^+^ T cell dysfunction trajectory (for **b**, **c** and **d**, *n* = 6,752 cells). Source data

**Extended Data Fig. 4:**
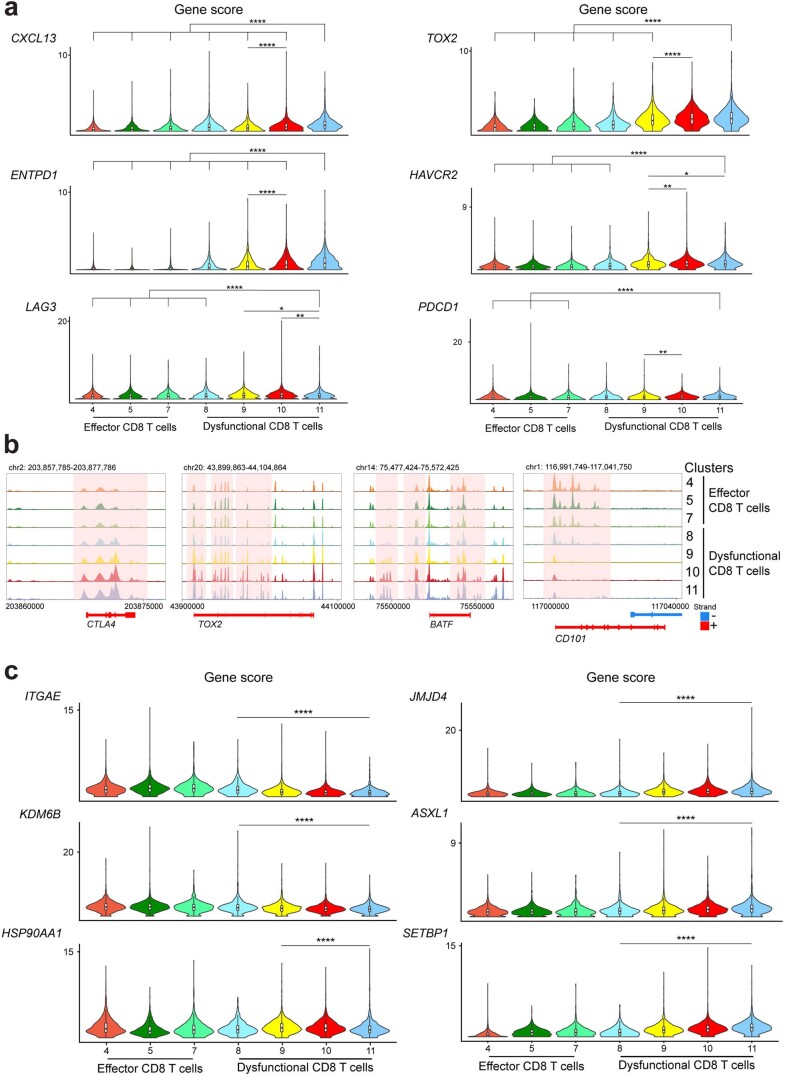
Regulatory chromatin landscapes of T cells in ccRCC. **a**, Violin plots of gene activity scores of the indicated genes for effector/non-dysfunctional (C4,5,7_CD8) and dysfunctional (C8,9,10,11_CD8) T cell clusters. Pair-wise comparisons of gene activity scores for the indicated gene between specified T cell clusters were determined using a two-sided Wilcoxon rank-sum test. Resulted p values further underwent multi-test correction with the FDR method (*, adjusted-p < 0.05; **, adjusted-p < 0.01; ****, adjusted-p < 0.0001; raw adjusted p values are listed in the Source Data file). **b**, Genome tracks of aggregate scATAC-seq data visualization of the indicated gene locus, clustered as denoted in Fig. 1b. **c**, Violin plots of gene activity scores of the indicated genes for dysfunctional (C8,9,10,11_CD8) T cell clusters (two-sided Wilcoxon rank sum test; ****, p < 0.0001; raw adjusted p values are listed in the Source Data file). Comparisons based on Fig. 2c (early/middle versus late dysfunction fate). Effector/non-dysfunctional (C4,5,7_CD8) T cell clusters are included for comparison. **a**, **c**, for clusters 4, 5, 7, 8, 9, 10, 11, n = 1810, 1821, 873, 924, 1775, 2440, 1613 cells, respectively. Source data

In contrast to most other solid tumors that respond to immunotherapy, in RCC high levels of infiltration by T cells is associated with inferior prognosis^21^. To gain insight into the heterogeneity of the fate composition of T cells, we further investigated the chromatin landscape of T cells in the kidney. Compared with clusters characterized by a high (>50%) contribution of effector CD8^+^ T cells from tumor tissue (C4,5,7_CD8), cells in C8,9,10,11_CD8, which were mainly populated with cells from tumors (Extended Data Fig. 2c), exhibited markedly higher gene accessibility for dysfunction-related genes (Figs. 1d and 2a and Extended Data Fig. 4a,b). Also, the *CD101* locus was more accessible in effector CD8^+^ T cells (Extended Data Fig. 4b), in contrast to other tumor models^11^ where this receptor has been associated with dysfunction.

To further dissect the mechanisms that drive the epigenomic states of CD8^+^ T cell dysfunction in ccRCC, we reconstructed a cellular trajectory that approximated the development of cells in C8,9,10,11 (refs. ^27,28^) and ordered these cells in pseudotime. This analysis identified a trajectory starting with C8, progressing through C9 and C10, and ending in C11 (Fig. 2b). We further identified genes with dynamic accessibility patterns across the trajectory (Fig. 2c). Genes that were highly accessible early in the trajectory included genes encoding tissue-resident memory T cells markers (*ITGAE*, *CXCR6*), costimulatory molecules (for example, *TNFSF14*) and effector-related molecules (for example, *TNF*) (Fig. 2c and Extended Data Fig. 4c). By contrast, genes that were accessible late in the trajectory included dysfunction-related markers (for example, *TOX*, *TOX2*, *CD38*, *PRDM1*, *ENTPD1*, *BTLA*, *CXCL13* and *CTLA4*) (Fig. 2c). In addition, we observed differential accessibility of genes encoding epigenetic modifiers along the trajectory, with high accessibility early in the trajectory for epigenetic modifiers such as *KDM6B* and later in the trajectory for modifiers including *JMJD4*, *SATB1*, *SMARCA2*, *SETBP1*, *STAG*2 and *ASXL1* (Fig. 2c and Extended Data Fig. 4c). Our data reveal a complex interplay of epigenetic modifiers during the progression through the dysfunction fates of CD8^+^ T cells in ccRCC. Moreover, we observed elevated accessibility of genes involved in protein homeostasis (proteostasis; for example, *HSPA2*, *HSPA1B*, *HSP90AA1*, *HSPH1*, *FBXO2* and *ATG7*) in the middle-to-late stages of dysfunction (Fig. 2c and Extended Data Fig. 4c). Thus, CD8^+^ T cell progression along the dysfunction path in ccRCC is accompanied by rewiring of the regulatory landscape of stress response genes. Consistent with these observations, regulatory elements within dysfunction-related clusters were enriched in gene ontology terms for biological process related to both chromatin remodeling and stress response (Extended Data Fig. 2f).

Having cataloged the chromatin landscape of the fates of T cells in the kidney, we next sought to identify the key TFs that could regulate these programs. Examination of TF-binding motif enrichment revealed distinct patterns between effector cells in C4,5,7_CD8 and dysfunctional cells in C9,10,11_CD8 (Fig. 1f). C4_CD8, which was composed of cells mainly from tumor and normal adjacent kidney tissues (Extended Data Fig. 2c), was associated with high activity of TFs involved in cytokine responses such as STAT3 and STAT5B (Extended Data Fig. 3b). Within C4_CD8, we observed tissue of origin specificity for TF activity. CD8^+^ T cells primarily derived from malignant tissue displayed binding motif enrichment for AP-1 complex members including FOS, FOSB, JUN, JUNB, JUND, MAFF, MAFG, MAFK and JDP2, which act downstream of T cell receptor (TCR) signaling to promote cell-cycle progression and effector functions including transcriptional activation of *IL2* (Fig. 1c and Extended Data Fig. 3b). In addition, IRF4, which cooperates with BATF/JUN heterodimers to promote CD8^+^ T cell effector differentiation^37^, showed increased TF activity in T cells prevalent in malignant tissue (Fig. 1c and Extended Data Fig. 3b). By contrast, CD8^+^ T cells with epigenetic landscapes similar to those from normal tumor-adjacent kidney tissue were characterized by binding motif enrichment for KLF2, a transcription factor involved in T cell quiescence^38^, and several less-described TFs including NFYA, SP4 and PBX3 (Fig. 1c and Extended Data Fig. 3b). Tumor-infiltrating CD4^+^ T cells (C12_CD4) were associated with binding motif enrichment for cluster-specific TFs such as members of the POU domain family (POU2F1, POU2F2 and POU2F3) (Extended Data Fig. 3b), in addition to TF programs shared with dysfunctional CD8^+^ T cells (Fig. 1f).

To specifically illuminate the repertoire of TFs underlying dysfunction-related chromatin configurations, we further examined the dynamic TF motif enrichment along the C8,9,10,11_CD8 dysfunction trajectory (Fig. 2b). We observed TFs such as TBX5 with enriched binding motifs throughout the dysfunction path compared with effector cells, with the highest enrichment for late dysfunction fates (Extended Data Fig. 3c). Previous studies showed that the TBX5-binding motif was enriched in loci more accessible in stem-like versus dysfunctional CD8^+^ T cells during chronic infection^39^. Our findings indicate that disease-specific regulatory processes may underlie T cell dysfunction in ccRCC patients. In addition, we detected dysfunction-stage-specific motif enrichment of TFs; for instance, IRF1 showed high activity early in the trajectory (Extended Data Fig. 3c), whereas NFAT (NFATC2, NFATC3) were more enriched late in the trajectory (Extended Data Fig. 3c). In line with previous reports showing that activation of NFAT in the absence of its binding partners JUN and FOS induces exhaustion^40^, our findings demonstrate enrichment of NFAT and JUN/FOS binding motifs in dysfunctional (C9,10,11_CD8) (Extended Data Fig. 3c) and effector (C4_CD8) clusters (Extended Data Fig. 3b), respectively. We also observed enriched TF activity for EPAS1 (also known as HIF2α) in C11_CD8 (Extended Data Fig. 3c).

We hypothesized that a dynamic TF regulatory program might underlie the progression from early to late dysfunction of tumor-infiltrating CD8^+^ T cells. To test this hypothesis, we examined dynamic changes in TF-binding motif enrichment along the trajectory of dysfunction (Fig. 2d). We observed diminishing binding motif enrichment for members of the ETS family (for example, ETS1, ETV2, ERG, ERF, FLI1, ELF1 and ELK1) during the progression from early- to late-stage dysfunction (Fig. 2d). Given that ETS factors have an important role in T cell homeostasis^41^, further investigation is needed to characterize the pathways modified by altered binding of ETS transcription factors during dysfunction progression. Late dysfunction stage was accompanied by motif enrichment for TFs previously found to be associated with T cell dysfunction, including NR4A, BATF and EOMES (Fig. 2d). In addition, we detected binding motif enrichment in the late dysfunction stage for TFs with unknown role in antitumor immunity; these included NFAT5, which has previously been implicated in the cellular response to osmotic stress^42^, as well as HIVEP1/3, NR2F2 and members of the T-box family (TBX10, TBR1) (Fig. 2d). Thus, our data indicate the existence of an elaborate network of TFs underlying the potential regulation of dysfunction fates of tumor-infiltrating T cells in ccRCC.

Recent single-cell RNA sequencing (scRNA-seq) profiling of immune cells in ccRCC patients showed that inhibitory interactions between dysfunctional CD8^+^ T cells and tumor-associated macrophages in advanced disease were associated with worse prognosis^22,23,25^. We attempted to further characterize the epigenetic landscape of myeloid cells (clusters 8 and 17; Extended Data Fig. 1b, c) in stage I ccRCC patients. However, at this early disease stage, we observed myeloid clusters predominantly composed of cells from blood and normal adjacent kidney tissue (Extended Data Fig. 1e), and the low numbers of cells (2,799 in total; 229 derived from tumors) precluded further characterization of the fate evolution of this cell type.

### Transcriptional programs of T cell dysfunction in ccRCC

**Fig. 3:**
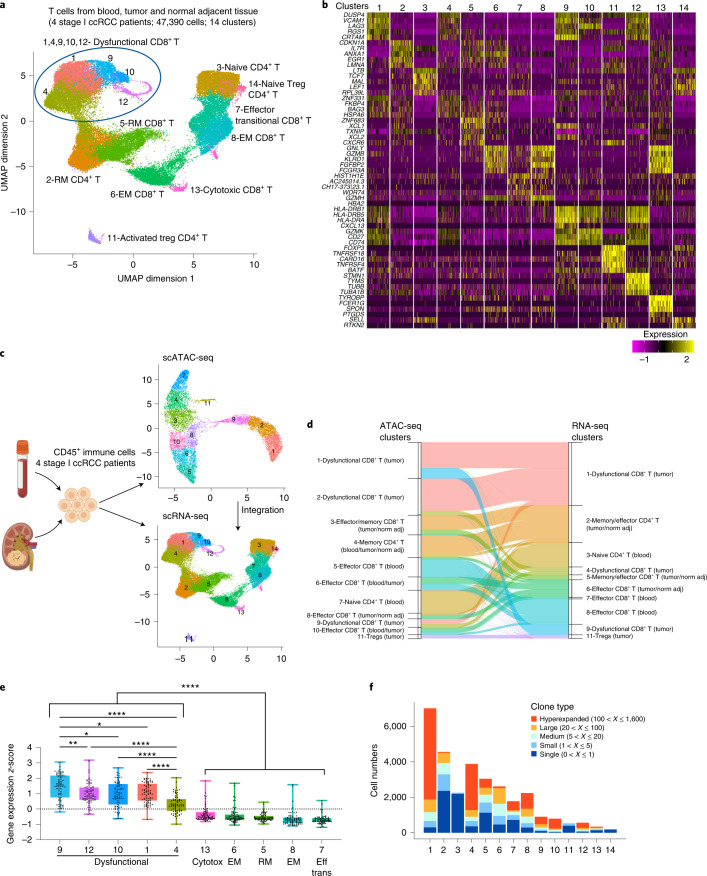
Single-cell transcriptional profiling of T cells in ccRCC. **a**, UMAP projection of 47,390 scRNA-seq profiles of T cells isolated from peripheral blood, tumor and adjacent normal tissue from four patients (a subset of the patients that were profiled epigenetically). Each dot corresponds to one single cell colored according to cell cluster. **b**, Heatmap of normalized expression of the top five marker genes in each cluster. **c**, Workflow for integrating scATAC-seq and scRNA-seq data from the same samples, divided into two. Left, experimental workflow. Image created with BioRender.com. Right, UMAP projection of scATAC-seq (top panel) and scRNA-seq (bottom panel) cells from four ccRCC patients for whom matched data from both modalities were available. **d**, Alluvial plot depicting annotation of cells in scATAC-seq data with cluster identities transferred from scRNA-seq data. The ribbon width corresponds to the number of cells in the specified scATAC-seq cluster (left side of the ribbon) that were annotated with the cluster identity of the corresponding scRNA-seq cluster (right side of the ribbon). To reduce clutter, only ribbons with width representing more than 20 cells are presented in this plot. For a complete overview, see Extended Data Fig. 6d. Color of ribbons corresponds to the color of the scRNA-seq clusters in the UMAP projection in **c**. **e**, Dysfunction gene expression levels in the indicated CD8^+^ T cell clusters (*n* = 4 patients; Methods). Each dot represents one of the 75 genes in the dysfunction gene module identified through gene module analysis; gene expression *z*-score distribution of these genes is depicted in boxplot format for each of the T cell clusters. One-way ANOVA test, Holm–Sidak correction for multiple comparisons. *P < 0.05, **P < 0.01, ****P < 0.0001. **f**, Plot showing counts of cells assigned into specific TCR clonal frequency ranges in each cluster as indicated in **a**. Source data

**Extended Data Fig. 5:**
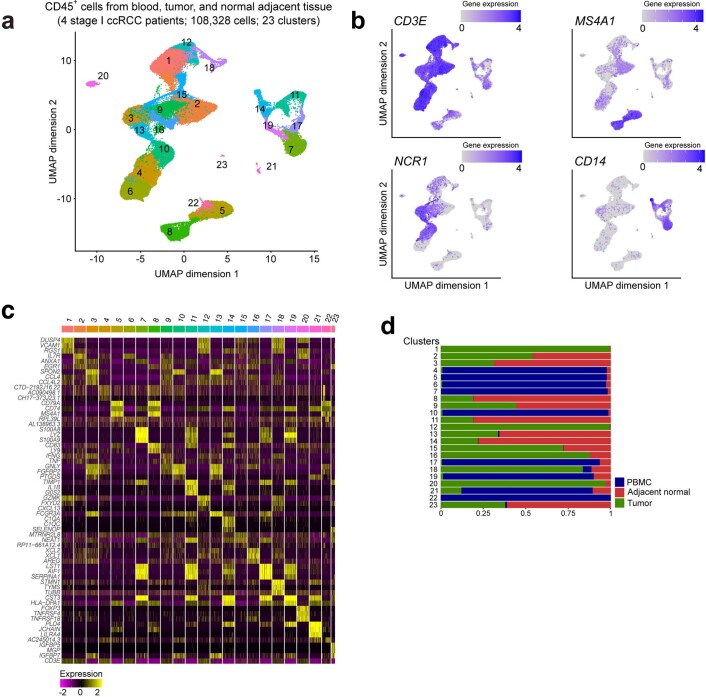
Single-cell transcriptional profiling of CD45^+^ cells in ccRCC. **a**, UMAP projection of 108,328 scRNA-seq profiles of CD45^+^ cells isolated from tumor, adjacent normal tissue, and peripheral blood of four patient samples. Each dot corresponds to one single-cell colored according to cell cluster. **b**, UMAP projection of cells colored by gene expression levels of selected lineage marker genes across CD45^+^ cell clusters. **c**, Heatmap of expression of the top 3 marker genes in each cluster across all clusters. **d**, Tissue-specific contribution of cells in each cluster derived from **a**. Source data

**Extended Data Fig. 6:**
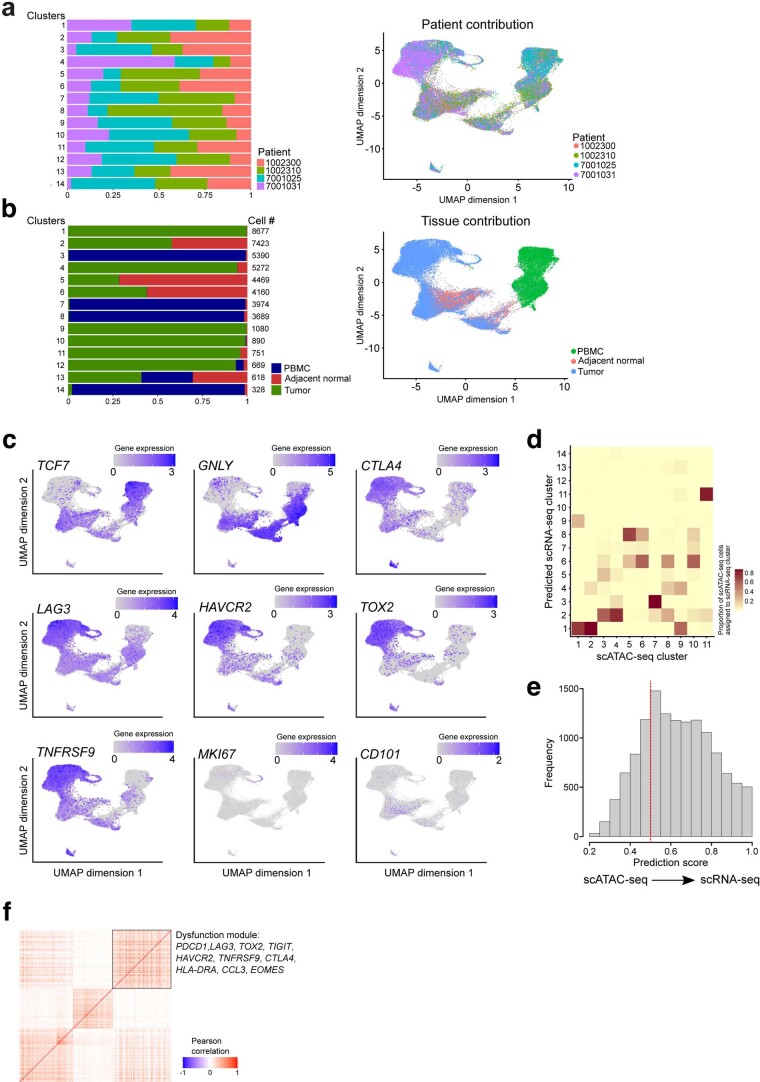
Single-cell transcriptional profiling of T cells in ccRCC. **a**, Patient-specific contribution of T cells in each cluster derived from Fig. 3a (left), and UMAP projection (right). **b**, Tissue-specific contribution of T cells in each cluster derived from Fig. 3a and UMAP projection (right). **c**, UMAP projection of cells colored by gene expression levels of the indicated marker genes. **d**, Heatmap showing the proportions of cells from each scATAC-seq cluster (x axis) that are annotated with cluster labels transferred from scRNA-seq clusters (y axis). **e**, Histogram of maximum prediction scores for every cell in scATAC-seq data; maximum prediction score measures the confidence level of annotating the scATAC-seq cell with cluster labels transferred from scRNA-seq data. Here, prediction score higher than 0.5 is considered of high confidence (n = 18736 cells). **f**, Gene-gene correlation heatmap of genes within each module identified by weighted gene co-expression module analysis. Selected genes from the dysfunction module (boxed) are listed (see Methods). Source data

To investigate the link between chromatin landscapes and transcriptional programs, we performed scRNA-seq in immune cells (CD45^+^) of a subset (*n* = 4) of the ccRCC patients that had been profiled epigenetically for which we had a sufficient number of CD45^+^ cells. Unsupervised clustering of 108,328 CD45^+^ cells revealed an immune cell composition of 23 clusters (Extended Data Fig. 5a,b,c). In line with our epigenetic profiling data, the myeloid clusters were predominantly composed of cells from blood and normal tumor-adjacent kidney tissue (Extended Data Fig. 5d). To gain insight into the diversity of gene expression programs of T cells in ccRCC, we performed fine clustering of the T cell subsets. A total of 14 clusters emerged, including ten clusters for CD8^+^ and four clusters for CD4^+^ T cells (Fig. 3a). Overall, the majority of the T cell transcriptional states identified were shared among patients (Extended Data Fig. 6a). The expression of known functional markers suggested that CD8^+^ T cell clusters included transitional (or early) effector (low *TCF7*, *IL7R*, *GNLY* and *GZMB* expression), resident memory (marked by high *ZNF683*, *CXCR6*, *IL7R* and *ANXA1* expression), effector memory (EM, marked by *GNLY*, *GZMB*, *IL7R* and *ANXA1* expression) and dysfunctional T cells, as well as conventional CD4^+^ T cells (naive, memory, effector) and CD4^+^ Tregs (Fig. 3a,b). T cells in these clusters exhibited distinct distributions of tissues of origin (Extended Data Fig. 6b). For example, C3_RNAseq, representing naive CD4^+^ T cells, was characterized by high expression of *TCF7* and *LEF1* and was almost exclusively populated with cells from blood, whereas cells in the effector T cell clusters C6_CD8_RNAseq and C7,8_CD8_RNAseq expressed high levels of *GNLY* and originated mainly from tumor or normal adjacent kidney tissue and blood, respectively (Fig. 3b and Extended Data Fig. 6b,c). Focusing on dysfunctional CD8^+^ T cells, we observed that cells in C1,4,9,10,12_CD8_RNAseq, predominant in tumors, expressed high levels of multiple dysfunction-related genes including *CTLA4*, *LAG3*, *HAVCR2* (*TIM3*) and *TOX2* (Extended Data Fig. 6b,c). In line with previous reports^24^, we found that in RCC, *TNFRSF9* (*4-1BB*) was mainly co-expressed with dysfunction-related markers (Extended Data Fig. 6c). In agreement with the epigenetic profiling data, *CD101* expression was detected, at low levels, outside the dysfunction clusters (Extended Data Fig. 6c).

To better understand how epigenetic and transcriptomic changes may regulate T cell fates in ccRCC, we integrated the derived gene activity scores with gene expression in four patients for whom matched data from both modalities were available^43,44^ (Fig. 3c). The clustering and annotation of scATAC-seq cell clusters were concordant between the subset of four patients and the total of eight ccRCC patients (Fig. 1b and Fig. 3c). We identified anchors between scATAC-seq and scRNA-seq data and annotated scATAC-seq cells via cell cluster labels transferred from scRNA-seq data^44^. We found that the majority (71%) of cells in the scATAC-seq dataset could be annotated via label transfer from scRNA-seq with confidence (maximum prediction score > 0.5; Fig. 3d and Extended Data Fig. 6d,e). Importantly, we found that multiple scATAC-seq T cell clusters with different chromatin states (C1,2,9_CD8_ATACseq) were annotated with the identity of one major dysfunction-associated scRNA-seq T cell cluster (C1_CD8_RNAseq; Fig. 3d). Thus, cells in certain dysfunction states, defined by transcriptomic analysis, may be destined for different fates as revealed by the epigenetic analysis. In line with previous reports^45^, we were not able to annotate a cluster of cycling cells in the scATAC-seq dataset through integration analysis, whereas there was a single cycling population in the scRNA-seq data (cluster 12; Fig. 3d and Extended Data Fig. 6c,d). These findings suggest that chromatin accessibility makes a limited contribution to changes in expression of cell-cycle-associated genes.

To examine whether CD8^+^ T cells exhibited a gradient of dysfunction states at the transcriptional level, similar to that observed at the epigenetic level, we performed weighted gene co-expression network analysis (WGCNA) on T cell scRNA-seq data and identified multiple sets of co-regulated gene modules, including a module associated with T cell dysfunction (Extended Data Fig. 6f). Based on the module analysis, for every gene within the dysfunction module we calculated the expression *z*-score across all T cell clusters to compare dysfunction gene activities. We then performed a quantitative comparison of the dysfunction levels across CD8^+^ T cell clusters and observed a spectrum of transcriptional intensity for dysfunction (Fig. 3e).

**Extended Data Fig. 7:**
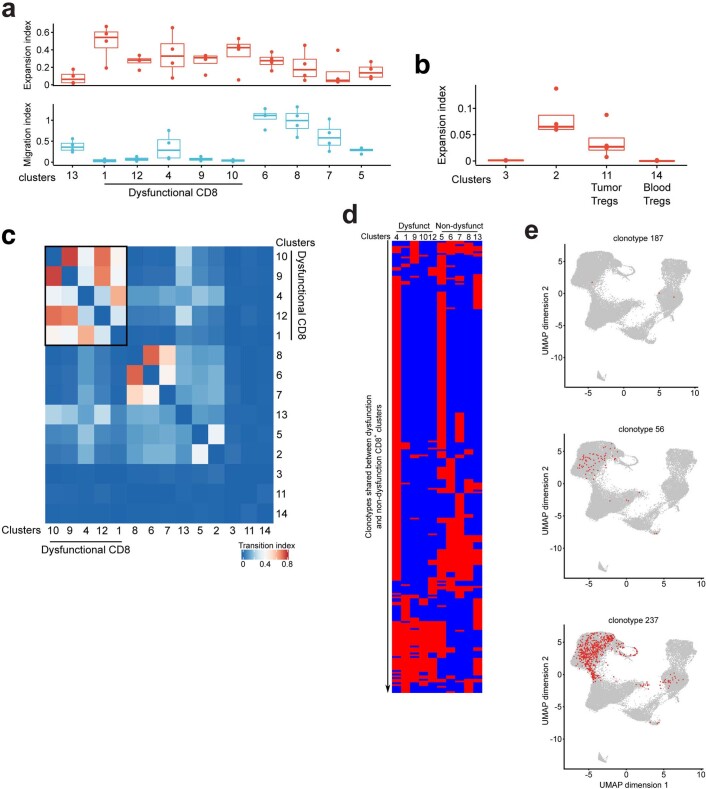
Single-cell TCR profiling of T cells in ccRCC. **a**, Clonal expansion and tissue migration indices of CD8^+^ T cell in the indicated clusters, calculated by STARTRAC. Each dot represents one patient; n = 4 patients. Box plot statistical values including whiskers, quartiles, median, max and min values are listed in the Source Data file. **b**, Clonal expansion index of the indicated CD4^+^ clusters, calculated by STARTRAC. Each dot represents one patient; n = 4 patients. Box plot statistical values including whiskers, quartiles, median, max and min values are listed in the Source Data file. **c**, Heatmap displaying the pair-wise cellular state transition index of T cells between clusters based on clonotype similarity, calculated by STARTRAC. **d**, Heatmap of 258 clonotypes (each row) shared by at least one of the dysfunction-related clusters (cl.1, 4, 9, 10, 12) and one of the non-dysfunctional CD8^+^ T cell clusters (cl.5, 6, 7, 8, 13). Red grid indicates detection of the clonotype in the corresponding cluster (each column) and blue grid indicates no detection of the clonotype in the corresponding cluster. **e**, Examples of clonotypes and their distribution on UMAP projection of T cells. Each red dot represents cells sharing the indicated clonotype. Source data

To investigate the connection between the identified functional T cell subsets and clonality, we used single-cell TCR sequencing (TCR-seq) data to track the lineage of each single T cell. We obtained 30,708 T cells with paired full-length TCR alpha and beta chains spanning the 14 clusters. We found 8,550 unique clonotypes, of which 1,512 were expanded clonotypes shared by at least two cells. To unveil the dynamic relationships of the TCR repertoire in ccRCC, we measured different indices based on the single T cell analysis by RNA-seq and a TCR tracking (STARTRAC) method^6^. First, focusing on CD8^+^ T cells, STARTRAC expansion (expa index) analysis revealed the dysfunctional cells (C1,4,9,10,12) to be in the clusters with the highest degree of clonal expansion (Extended Data Fig. 7a and Fig. 3f). Of the two Treg clusters, C14_Treg-Naive was predominantly composed of cells from peripheral blood, whereas C11_Treg-Activated cells were predominantly from tumor tissue (Extended Data Fig. 6b). We found that Treg clonal expansion was cluster specific and occurred within tumors (Extended Data Fig. 7b), suggesting the potential for tumor-associated antigen recognition and local clonal expansion of suppressive tumor-resident Tregs. To evaluate the extent of tissue migration across blood, normal tissue and malignant kidney tissue of a certain clonotype, we performed STARTRAC migration (migr index) analysis. We observed that T effector memory (T_EM_) and T effector (T_Eff_) cells were associated with the highest mobility across tissues, whereas dysfunctional CD8^+^ T cells exhibited tumor specificity (Extended Data Fig. 7a). Finally, we tested the extent of state transition of each clonotype among T cell clusters. STARTRAC transition (tran index) analysis indicated that cells from the dysfunction clusters (C1,4,9,10,12_CD8) were connected, corroborating the developmental trajectory of dysfunction observed at the chromatin and gene expression levels (Extended Data Fig. 7c). Taken together, our findings allow us to link the transcriptional states of T cells in treatment-naive patients to TCR clonality and provide a resource for investigating T cell dynamics in ccRCC.

### NF-κB induces a pro-apoptotic program in dysfunctional T cells

**Fig. 4:**
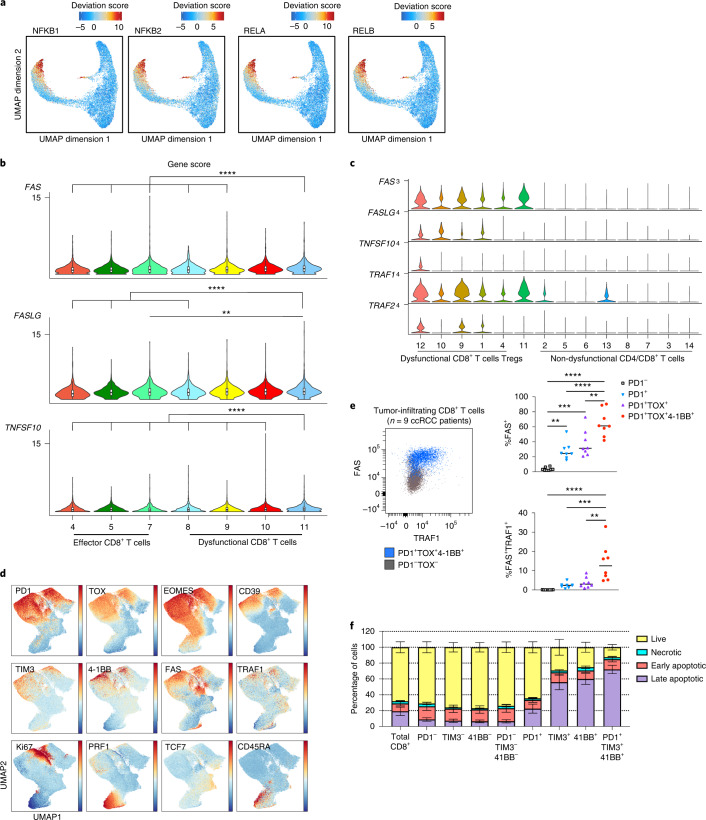
NF-κB drives a pro-apoptotic program in late dysfunctional CD8^+^ T cells infiltrating ccRCC. **a**, UMAP projection of scATAC-seq profiles colored by chromVAR TF motif bias-corrected deviations of the indicated TFs (NFKB1/2 and RELA/B were significantly enriched in C11_CD8 compared with the other CD8 T cell clusters; two-sided Wilcoxon rank-sum test *P* < 2.2 × 10^−16^, *n* = 18,736 cells). **b**, Violin plots of gene activity scores of the indicated NF-κB transcriptional targets for effector/nondysfunctional (C4,5,7_CD8) and dysfunctional (C8,9,10,11_CD8) T cell clusters. Pairwise comparisons of gene activity scores for the indicated gene between specified T cell clusters were determined using a two-sided Wilcoxon rank-sum test. The resulting *P* values underwent multi-test correction with the FDR method (**adjusted *P* < 0.01, ****adjusted-*P* < 0.0001; raw adjusted *P* values are listed in the source data; for clusters 4, 5, 7, 8, 9, 10, 11, *n* = 1,810, 1,821, 873, 924, 1,775, 2,440 and 1,613 cells, respectively). **c**, Violin plots showing gene expression levels of the indicated NF-κB transcriptional targets in CD4^+^ and CD8^+^ clusters, clustered as indicated in Fig. 3a (for clusters 1, 2, 3, 4, 5, 6, 7, 8, 9, 10, 11, 12, 13 and 14, *n* = 8,677, 7,423, 5,390, 5,272, 4,469, 4,160, 3,974, 3,689, 1,080, 890, 751, 669, 618 and 328 cells, respectively). **d**, Flow cytometry analysis of tumor-infiltrating CD8^+^ T cells. UMAP projection of expression of selected T cell markers in CD8^+^ T cells (*n* = 9 ccRCC patients). Color gradient indicates expression level (red, high; blue, low). **e**, Protein expression of NF-κB targets in tumor-infiltrating CD8^+^ T cells. FACS plot (FAS versus TRAF1) overlay of PD-1^−^TOX^−^ (nondysfunctional) and PD-1^+^TOX^+^4-1BB^+^ (late dysfunctional) CD8^+^ T cell populations (left). Dot plots showing the percentages of FAS^+^ and FAS^+^TRAF1^+^ cells in different CD8^+^ T cell populations (right; one-way ANOVA test, Holm–Sidak correction for multiple comparisons; *n* = 9 ccRCC patients; **P < 0.01, ***P < 0.001, ****P < 0.0001; raw *P* values are listed in the source data). **f**, Apoptosis flow cytometry analysis. Bar graph showing the viability status (mean ± s.e.m.) as defined by flow cytometry for the indicated CD8^+^ T cell subsets (*n* = 10 ccRCC patients; raw *P* values are listed in the source data). Source data

**Extended Data Fig. 8:**
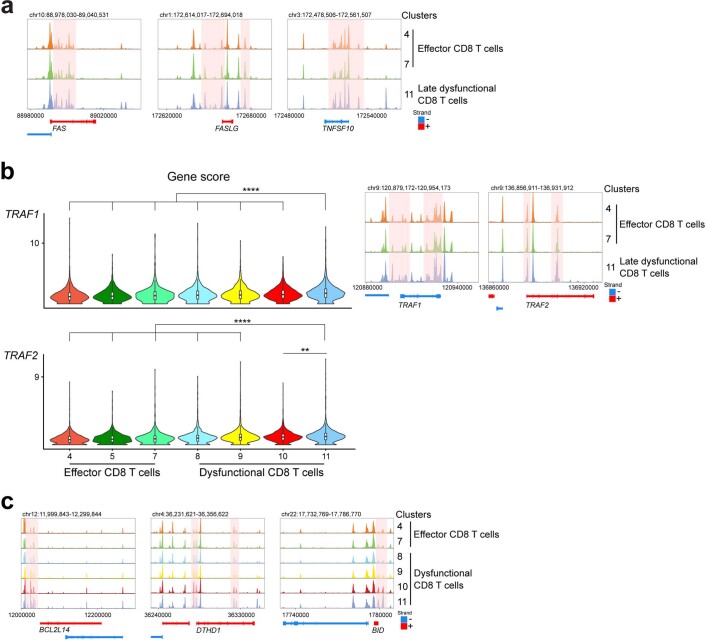
*Cis*-regulatory element accessibility of proapoptotic genes in late dysfunctional ccRCC infiltrating CD8^+^ T cells. **a**, Genome tracks of aggregate scATAC-seq data visualization of NFκB transcriptional targets gene loci, clustered as indicated in Fig. 1b. **b**, Violin plots of gene activity scores (left) and genome tracks of aggregate scATAC-seq data visualization of the NFκB transcriptional target genes (right), clustered as indicated in Fig. 1b. Pair-wise comparisons of gene activity scores for the indicated gene between specified T cell clusters were determined using a two-sided Wilcoxon rank-sum test. Resulted p values further underwent multi-test correction with the FDR method (**, adjusted-p < 0.01; ****, adjusted-p < 0.0001; raw adjusted p values are listed in the Source Data file; for clusters 4, 5, 7, 8, 9, 10, 11, n = 1810, 1821, 873, 924, 1775, 2440 and 1613 cells, respectively). **c**, Genome tracks of aggregate scATAC-seq data visualization of proapoptotic gene loci, clustered as indicated in Fig. 1b. Source data

TCR signaling upon tumor antigen-dependent engagement results in activation and nuclear translocation of several TFs including NF-κB, to promote survival and effector differentiation^46^. Ostensibly at odds with the critical role of NF-κB in T cell activation, our epigenomic analysis revealed robust NF-κB motif enrichment in late dysfunctional (C11_CD8) RCC-infiltrating CD8^+^ T cells (Fig. 1f). In addition to NFKB1 and NFKB2, we observed increased TF activity for other members of the NF-κB family, indicating enriched activity of both classical and alternative NF-κB pathways in late dysfunctional CD8^+^ T cells (Fig. 4a). Moreover, the regulatory elements in the cluster composed of late dysfunctional CD8^+^ T cells were enriched in gene ontology terms for biological processes related to NF-κB pathway activity (Extended Data Fig. 2f). Previous studies have shown that in chimeric antigen receptor (CAR) T cells, self-clustering of CD19 CAR results in 4-1BB-dependent persistent activation of the NF-κB pathway, upregulation of pro-apoptotic genes and apoptosis of CAR T cells^47^. A pro-apoptotic role of NF-κB in T cells in diverse contexts such as infection has also been described^46^. We hypothesized that in late dysfunctional CD8^+^ T cells in ccRCC, NF-κB could drive a pathway that impairs survival. To test this hypothesis, we monitored the chromatin landscape of genes involved in apoptotic pathways. We first examined *FAS* (*CD95*), *FASLG* and *TNFSF10* (*TRAIL*), which have been shown to be direct transcriptional targets of NF-κB^48,49^. We observed significantly increased gene scores for *FAS*, *FASLG* and *TNFSF10* in C11_CD8 compared with the nondysfunctional/effector tumor-infiltrating CD8^+^ T cells and earlier dysfunction fates (Fig. 4b). For a number of potential enhancers and/or regulatory regions of those genes, we detected increased accessibility along the axis from nondysfunction to late dysfunction (Extended Data Fig. 8a). Moreover, we observed significantly increased gene scores and accessibility of potential regulatory elements for *TRAF1* and *TRAF2* in the late dysfunction cluster compared with effector cells and earlier dysfunction fates (Extended Data Fig. 8b). *TRAF1* and *TRAF2* are NF-κB-inducible genes encoding adapter molecules that function upstream of NF-κB and downstream of diverse signaling pathways, including the 4-1BB signaling pathway. In complete agreement with our epigenetic profiling data, gene expression of *FAS*, *FASLG*, *TNFSF10, TRAF1* and *TRAF2* was higher in the clusters corresponding to dysfunctional tumor-infiltrating CD8^+^ T cells (Fig. 4c). To further investigate the potential link between high NF-κB pathway activity in dysfunctional T cells and cell death, we examined the chromatin landscape of other pro-apoptotic genes including *BCL2L14*, *DTHD1* and *BID* and found increased accessibility for potential regulatory elements of those genes along the nondysfunction to late dysfunction axis (Extended Data Fig. 8c). Moreover, GREAT gene ontology analysis of regulatory elements enriched in late dysfunctional CD8^+^ T cells confirmed enrichment in biological processes related to apoptosis (Extended Data Fig. 2f).

TCR repertoire analysis across blood, tumor tissue and normal adjacent kidney tissue revealed potential sources of CD8^+^ T cells that enter the dysfunction trajectory^50^. Among the 8,550 unique clonotypes, we identified 258 clonotypes present in at least one of the dysfunction-related clusters and one of the nondysfunctional CD8^+^ T cell clusters (Extended Data Fig. 7d). We observed clonotypes shared between blood (effector memory) and dysfunctional CD8^+^ T cell clusters (for example, clonotype 187), normal adjacent tissue/nondysfunctional tumor clusters (resident memory) and dysfunctional CD8^+^ T cell clusters (for example, clonotype 56), as well as among the three tissues (for example, clonotype 237) (Extended Data Fig. 7d,e).

**Extended Data Fig. 9:**
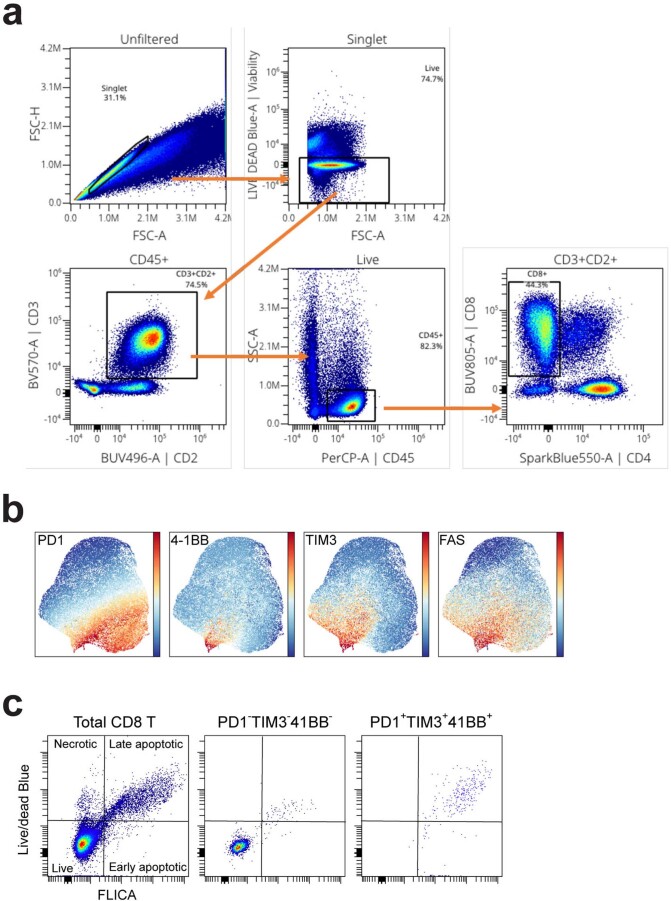
Apoptosis flow cytometry analysis. **a**, Gating strategy for flow cytometry analysis of tumor-infiltrating CD8^+^ T cells. **b**, UMAP projection of tumor-infiltrating CD8^+^ T cells from ccRCC patients (n = 5), colored by the expression levels of the indicated markers. Color gradient indicates expression level (red-high, blue-low). **c**, Representative viability FACS plots for the indicated CD8^+^ T cell subsets.

At the protein level, we used flow cytometry to validate the co-expression of dysfunction markers (for example, PD1, TOX, EOMES, CD39 and TIM3) with FAS, TRAF1 and 4-1BB in tumor-infiltrating CD8^+^ T cells in ccRCC patient samples that had also been analyzed by single-cell transcriptomic/epigenomic assays, as well as in an additional set of tumor samples (*n* = 9; Supplementary Table 1) (Fig. 4d and Extended Data Fig. 9a). As expected, these dysfunctional cells expressed low levels of proteins associated with effector function and memory (Fig. 4d). Notably, the protein expression of FAS and TRAF1 was significantly higher in late dysfunctional CD8^+^ T cells marked by co-expression of PD1, TOX and 4-1BB (Fig. 4e). To further investigate whether the late dysfunctional CD8^+^ T cells were prone to cell death as a consequence of chronic NF-κB activation in the tumor, we employed flow cytometry to monitor different stages of cell death (early/late apoptosis and necrosis). We utilized established markers of dysfunction (PD1 and TIM3), as well as 4-1BB, which is co-expressed with dysfunction markers in RCC^24^, and NF-κB transcriptional targets (FAS) to examine the relationship between dysfunctional state and cell death (Extended Data Fig. 9b). Phenotypic analysis revealed that CD8^+^ T cells with high expression levels of PD1, TIM3 and 4-1BB, corresponding to the late stage of dysfunction according to chromatin and gene expression data, showed the highest rates of late apoptosis (Fig. 4f and Extended Data Fig. 9c).

**Fig. 5:**
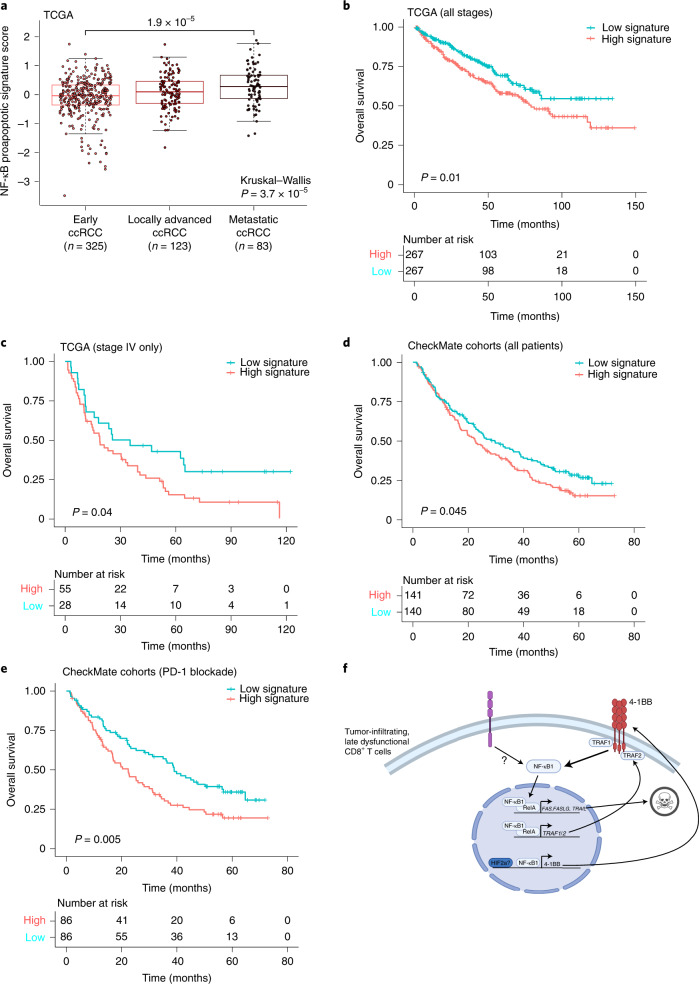
A chromatin-derived NF-κB signature of late dysfunctional CD8^+^ T cells predicts patient survival. **a**, A gene expression signature of pro-apoptotic NF-κB targets in late dysfunctional CD8^+^ T cells for the indicated disease stage in the external TCGA KIRC cohort (two-sided Wilcoxon rank-sum test for pairwise comparison; Kruskal–Wallis test for global *P* value; *n* = 325 patients for early ccRCC, *n* = 123 patients for locally advanced ccRCC, *n* = 83 patients for metastatic ccRCC). Box plot statistical values including whiskers, quartiles, and median, max and min values are listed in the source data. **b**, Overall survival for the overall TCGA ccRCC cohorts based on high pro-apoptotic NF-κB signature (≥median) versus low signature expression. Log-rank test was used to compare the survival between the two groups. *χ*^2^ = 6.6 on 1 degrees of freedom, *P* = 0.01. **c**, Overall survival for the advanced TCGA ccRCC cohorts based on high pro-apoptotic NF-κB signature (≥median) versus low signature expression. Log-rank test was used to compare the survival between the two groups. *χ*^2 ^= 4.3 with 1 degree of freedom, *P* = 0.04. **d**, Overall survival for the entire CheckMate cohort, based on high pro-apoptotic NF-κB signature (≥median) versus low signature expression. Log-rank test was used to compare the survival between the two groups. *χ*^2 ^= 4 with 1 degree of freedom, *P* = 0.045. **e**, Overall survival for the PD-1 blockade CheckMate cohort, based on high pro-apoptotic NF-κB signature (≥median) versus low signature expression. Log-rank test was used to compare the survival between the two groups. *χ*^2 ^= 7.7 for 1 degree of freedom, *P* = 0.005. **f**, Model of the NF-κB pro-apoptotic program in late dysfunctional CD8^+^ T cells. Image created with BioRender.com. Source data

**Extended Data Fig. 10:**
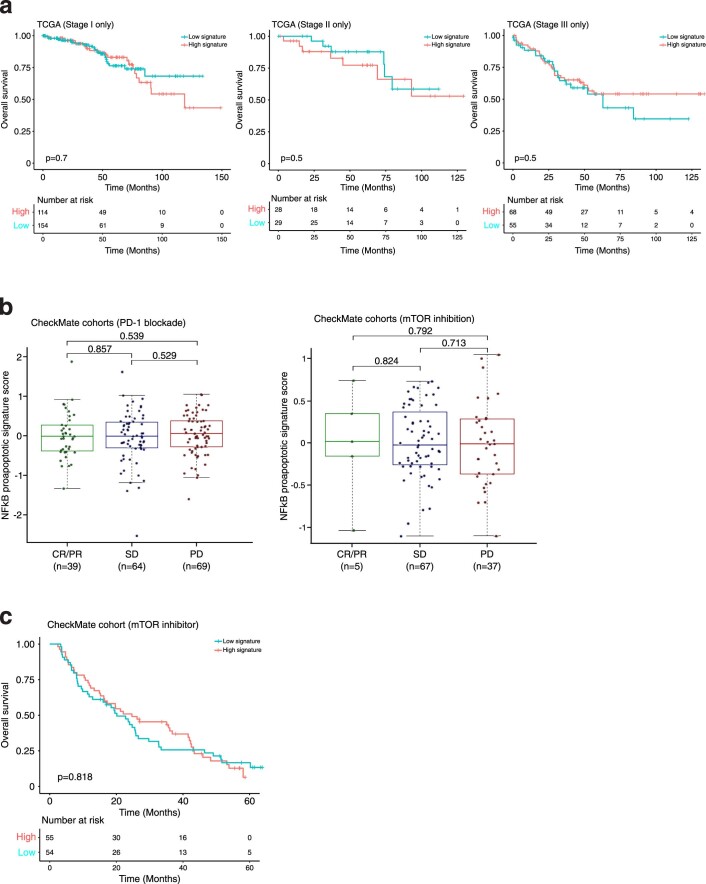
Single-cell chromatin derived signature and survival. **a**, Overall survival for the indicated early stage (I, II, III) TCGA ccRCC cohorts based on high proapoptotic NFκB signature (≥median) versus low signature expression. Log-rank test was used to compare the survival between the two groups in each panel. TCGA stage I: Chisq=0.1 on 1 degrees of freedom, p = 0.7; TCGA stage II: Chisq=0.4 on 1 degrees of freedom, p = 0.5; TCGA stage III: Chisq=0.5 on 1 degrees of freedom, p = 0.5. **b**, NFκB-derived signature analysis of tumors from the CheckMate cohorts of advanced ccRCC examining response (two-sided Wilcoxon rank-sum test) following treatment with PD-1 blockade (left) or mTOR inhibition (right). For PD-1 blockade, CR/PR, n = 39 patients, SD n = 64 patients, PD n = 69 patients. For mTOR inhibition, CR/PR, n = 5 patients, SD n = 67 patients, PD n = 37 patients. Box plot statistical values including whiskers, quartiles, median, max and min values are listed in the Source Data file. **c**, Overall survival for the mTOR inhibition CheckMate cohort, based on high proapoptotic NFκB signature (≥median) versus low signature expression. Log-rank test was used to compare the survival between the two groups in each panel; Chisq=0.1 on 1 degrees of freedom, p = 0.818. Source data

Finally, we tested whether the enriched NF-κB activity in late dysfunctional CD8^+^ T cells—specifically, the NF-κB-driven pro-apoptotic transcriptional program—was predictive of clinical outcome in human ccRCC. To evaluate this, we generated a signature composed of previously described cell-death-related NF-κB-inducible genes^47,48^ that in our dataset exhibited: increased regulatory element accessibility at the chromatin level, increased gene expression at the mRNA level and increased protein level in late dysfunctional CD8^+^ T cells compared with nondysfunctional/effector CD8^+^ T cells. Specifically, this signature included the pro-apoptotic genes *FAS*, *FASLG* and *TNFSF10*, as well as *TRAF1* and *TRAF2* (ref. ^51^). Notably, in the ccRCC cohort (KIRC) of The Cancer Genome Atlas (TCGA)^52^, expression of the pro-apoptotic NF-κB signature was increased in more advanced disease stages (*P* = 1.9 × 10^−5^; Fig. 5a). High expression of this gene signature was associated with reduced overall patient survival in the TCGA KIRC cohort (*P* = 0.01; Fig. 5b), and this association was specific in patients with advanced disease (stage IV; *P* = 0.04; Fig. 5c and Extended Data Fig. 10a). Next, we examined whether the pro-apoptotic NF-κB activity was associated with response to therapy. We evaluated the signature in patients treated with either nivolumab (PD-1 blockade) or everolimus (mTOR inhibitor) in the CheckMate RCC cohorts^15,53^. The pro-apoptotic NF-κB signature was not predictive of response to either PD-1 blockade or mTOR inhibition (Extended Data Fig. 10b). However, patients with high expression of this signature had worse overall survival (*P* = 0.045; Fig. 5d). Notably, this association was observed only among patients receiving nivolumab (*P* = 0.005; Fig. 5e and Extended Data Fig. 10c). These findings suggest that the ccRCC microenvironment induces a rewired, pro-apoptotic transcriptional program of NF-κB in a subset of tumor-infiltrating CD8^+^ T cells, which is associated with a worse overall prognosis.

## Discussion

In RCC, in contrast to other immunotherapy-responsive solid tumors, high infiltration by CD8^+^ T cells is associated with a worse prognosis^21^. To test the hypothesis that heterogeneity of infiltrating CD8^+^ T cell fate may, at least in part, explain this paradox, we performed deep epigenetic profiling at the single-cell level, along with scRNA/TCR-seq and flow cytometry of immune cells from treatment-naive ccRCC patients. Developmental fates of T cells in cancer patients are orchestrated by a complex interplay between epigenetic modulators and *cis*-regulatory sequences that drive gene expression programs in response to cues emanating primarily from the tissue of residency. Recent technological advances including ATAC-seq^27,34^ have proven valuable for functional genome annotation^54^.

Our findings provide a comprehensive map of the dynamic changes in the dysfunction-regulome of T cells, focusing on the path to dysfunction of ccRCC-infiltrating CD8^+^ T cells. We found a dynamic evolution of potential regulatory elements of epigenetic modulators that may, in part, explain the unique chromatin accessibility signature of exhausted T cells described previously in cancer and chronic infections^4,9–11^. We also found alterations in the accessibility of potential regulatory elements of stress response genes during progression of dysfunction, probably reflecting the extensive fate rewiring that occurs along the dysfunction axis. T cells acquiring a late dysfunction chromatin identity also displayed enriched binding motifs for HIF2α. The potential effect of HIF2α inhibitors^55,56^ on T cells needs further investigation. We also provide evidence suggesting stark differences between the path to T cell dysfunction in ccRCC and that in other tumor types or in chronic infection at the level of TFs (for example, TBX5) and surface markers (for example, CD101). Moreover, by integrating the derived gene activity scores with gene expression levels, we found that chromatin accessibility analysis could define dysfunction fates with high resolution.

The role of NF-κB as an antiapoptotic regulatory factor in diverse cell types has been previously described^46,51^. Strikingly, our studies showed that in late dysfunctional CD8^+^ T cells infiltrating ccRCC, NF-κB induced a pro-apoptotic program (Fig. 5f). Importantly, a gene signature composed of cell-death-related NF-κB-induced targets could predict patient survival in ccRCC. Previous studies have shown that 4-1BB is co-expressed with exhaustion markers^24^ and is upregulated during hypoxia^57^. Further studies are needed to generate mechanistic insights into the signaling pathways that converge on NF-κB in ccRCC-infiltrating CD8^+^ T cells and to test whether NF-κB-hijacking of a pro-apoptotic pathway is an immune feature of other cancers (Fig. 5f).

A notable limitation of our study was the epigenomic analysis of treatment-naive patient samples in the early disease stage (stage I). Further studies are needed to map the epigenetic landscape during disease progression and in relation to the genetic makeup^53,58,59^ and subsets of RCC^18,60^. Recent scRNA-seq studies have shown that terminally exhausted CD8^+^ T cells are enriched in advanced ccRCC and specific CD8^+^ T cell phenotypes are associated with immune-checkpoint blockade^22,23,25^. Investigation of the impact of therapy on the epigenetic landscape of immune cells and the plasticity of the TF networks in response to or resistance to immunotherapy is warranted. Profiling of epigenetic states may help us to understand whether T cell populations can be durably reactivated by therapy. Our results in ccRCC, along with previous findings in preclinical models^9–11^, raise questions regarding the potential clinical applicability of epigenetic therapy in preventing progression to or even reversing unfavorable epigenetic states. Alternatively, targeting key transcription factors with emerging therapeutic modalities may remodel the epigenome of dysfunctional immune cells. Finally, our analysis included a modest number of patient samples. Future work should enhance our study by profiling the epigenetic landscape of a larger number of patient samples and integrating epigenetic and transcriptomic data in an immune cell fate atlas for ccRCC.

In conclusion, our study provides a unique resource of single-cell epigenomic data along with transcriptomic, TCR- and protein-based information for immune cells in ccRCC patients. Our study demonstrates the power of single-cell epigenomics for the derivation of epigenetic and/or fate signatures with prognostic value, which represents a step toward immune-type-based patient stratification. These comprehensive single-cell maps of T cells could facilitate understanding of T cell biology in cancer patients and guide therapeutic strategies to overcome resistance due to immune cell fate heterogeneity.

## Methods

### Human specimens

Surgically removed stage I primary ccRCC tumor tissue, adjacent normal kidney tissue and whole blood were obtained within 24 h post-surgery (Avaden Bio; single-cell analyses). Additional RCC tumor samples (stage I; flow cytometry/functional assays) were obtained from Discovery Life Science. No patient had received prior systemic therapy for their cancer. No ethical approval was required for the study, as informed consent was obtained prior to tissue acquisition by the vendors, as stated by their policies. Patient information is summarized in Supplementary Table 1.

### Sample processing

Renal tumor and adjacent normal tissue samples were dissociated into single cells by a semi-automated mechanical and enzymatic process. Tumor tissue was cut into pieces of (~2–3 mm) and transferred to C Tubes (Miltenyi Biotech) containing a mix of enzymes (Tumor Dissociation Kit, human; Miltenyi Biotech). Mechanical dissociation was performed on a gentleMACS dissociator (program 37C_h_TDK_1). To allow for enzymatic digestion, the tubes were incubated for 30 min at 37 ^o^C, with rotation, after the first and second mechanical dissociation step. Mononuclear cells from whole peripheral blood of paired subjects were isolated by density gradient centrifugation using SepMate tubes (Stem Cell Technologies). Cells were then cryopreserved in Recovery Cell Culture Freezing Medium (Thermo Fisher). Prior to single-cell sequencing, cells were rapidly thawed in warm Dulbecco’s modified Eagle medium (Gibco) supplemented with 10% fetal bovine serum (FBS) and pelleted.

### Cell sorting

Tumor and adjacent normal tissue cells and peripheral blood mononuclear cells (PBMCs) were resuspended in FACS staining buffer (1% bovine serum albumin (BSA) and 1 mM EDTA in Dulbecco’s phosphate-buffered saline (PBS); Gibco) and incubated with Human TruStain FcX (BioLegend) for 10 min on ice to block nonspecific binding to Fc receptors. Cells were then washed and stained with CD45-PE-Dazzle594 (BioLegend) for 20 min on ice. Next, cells were filtered and resuspended in FACS staining buffer with addition of DNase for FACS sorting. DAPI was added to the cell suspension immediately before FACS sorting for dead cell exclusion. Live CD45^+^ single cells were sorted for downstream single-cell analysis.

### Nucleus isolation

Isolation of nuclear suspensions was performed according to ref. ^27^ and the demonstrated protocol: Nuclei Isolation for Single Cell ATAC Sequencing (10x Genomics). Owing to the limited numbers of immune cells from patient samples, we followed a low cell input nucleus isolation protocol. Briefly, cells were resuspended in 50 μl PBS + 0.04% BSA and transferred to a 0.2 ml tube and centrifuged (300*g* for 5 min at 4 ^o^C). The supernatant was removed, added to 45 μl of chilled lysis buffer (10 mM Tris-HCl (pH 7.4), 10 mM NaCl, 3 mM MgCl_2_, 0.1% Tween-20, 0.1% Nonidet P40 Substitute, 0.01% digitonin and 1% BSA) and gently mixed by pipetting. The tube was then incubated on ice for 3 min. After lysis, 50 μl of chilled wash buffer (10 mM Tris-HCl (pH 7.4), 10 mM NaCl, 3 mM MgCl_2_, 0.1% Tween-20 and 1% BSA) was added without mixing. Nuclei were centrifuged (500*g* for 5 min at 4 ^o^C) and the supernatant was carefully removed. Next, 45 μl of diluted Nuclei Buffer (10x Genomics) was added without mixing, nuclei were centrifuged (500*g* for 5 min at 4 ^o^C), and the supernatant was carefully removed. Isolated nuclei were resuspended in 7 μl chilled diluted Nuclei Buffer (10x Genomics). Nuclei were immediately used to generate scATAC-seq libraries as described in the Methods section.

### Preparation and sequencing of scATAC-seq library

Nuclei were suspended at 1:20 dilution in 20x Nuclei Buffer provided by the Chromium NextGEM Single Cell ATAC Library & Gel Bead Kit. Nuclei were aliquoted for the transposition reaction to target 2000 recovered nuclei. Transposed nuclei were partitioned using a Chromium Single Cell Instrument (10x Genomics) and libraries were generated. Sequencing was performed on an Illumina NextSeq 500 platform (Illumina) by a multiplexed paired-read run with 2×50 cycles. Cell Ranger ATAC v.3.0.2 (10x Genomics) was used to perform demultiplexing and read alignment.

### Preparation and sequencing of scRNA-seq and TCR library

Single cells suspended in PBS with 0.04% BSA were loaded on a Chromium Single Cell Instrument (10x Genomics). RNA-seq and V(D)J libraries were prepared using the Chromium Single Cell 5′ Library, Gel Beads, & Multiplex Kit (10x Genomics). After amplification, cDNA was split into RNA-seq and V(D)J library aliquots. To enrich the V(D)J library aliquot for TCR a/b, the cDNA was split into two 20 ng aliquots and amplified in two rounds using primers designed in-house. Specifically, for first-round amplification, the primers used were MP147 (ACACTCTTTCCCTACACGACGC) for short R1, MP120 (GCAGACAGACTTGTCACTGGA) for human TRAC and MP121(CTCTGCTTCTGATGGCTCAAACA) for human TRBC. For second round amplification, 20 ng aliquots from the first round were amplified using MP147, MP128 (GTGACTGGAGTTCAGACGTGTGCTCTTCCGATCTGCAGGGTCAGGGTTCTGGATA), a nested R2 plus human TRAC and MP129 (GTGACTGGAGTTCAGACGTGTGCTCTTCCGATCTGCAGGGTCAGGGTTCTGGATA), a nested R2 plus human TRABC. V(D)J libraries were prepared from 25 ng each of hTRAC- and hTRBC-amplified cDNA. Paired-end sequencing was performed on an Illumina NextSeq500 for RNA-seq libraries (Read 1: 26-bp for UMI and cell barcode, 8-bp i7 sample index; Read 2: 55-bp transcript read) and V(D)J libraries (Read 1: 150-bp, 8-bp i7 sample index; Read 2: 150-bp read). For RNA-seq libraries, Cell Ranger Single-Cell Software Suite (10X Genomics, v.2.2.0) was used to perform sample demultiplexing, alignment, filtering and UMI counting. The human GRCh38 genome assembly and RefSeq gene model for human were used for the alignment. For V(D)J libraries, Cell Ranger Single-Cell Software Suite (10x Genomics, v.3.0.2) was used to perform sample demultiplexing, de novo assembly of read pairs into contigs, alignment and annotation of contigs against the germline segment V(D)J reference sequences from IMGT, labeling and location of CDR3 regions and grouping of clonotypes.

### Flow cytometry staining

Single-cell suspensions were aliquoted into a 96-well V-bottomed plate. The plate was centrifuged (500 *g* for 3 min at 4 °C) and supernatant was removed. Cell pellets were washed with FACS buffer (PBS with 5% FBS and 2 mM EDTA) and resuspended in a mixture of TruStain FcX and True-Stain Monocyte Blocker (BioLegend) to block human Fc receptors and nonspecific binding, respectively, and LIVE/DEAD Fixable Blue Dead Cell Stain (Thermo Fisher) for 15 min on ice. Cells were washed, resuspended in a mixture of fluorochrome-conjugated cell-surface-staining antibodies diluted in FACS buffer and incubated on ice for 20 min. Cells were washed and resuspended in fixation/permeabilization buffer (Thermo Fisher) on ice for 20 min. After fixation and permeabilization, cells were washed twice with permeabilization/wash buffer. Cell pellets were resuspended in intracellular staining antibodies diluted in permeabilization buffer at 4 ^o^C overnight. To quantify apoptosis status, we used a Vybrant FAM-VAD-FMK poly caspase kit (Molecular Probes). Samples were filtered using an AcroPrep Advance Filter Plate and acquired using a BD Symphony A5 cytometry or Cytek Aurora spectral cytometer. The antibodies used in this study were as follows: PD-1 (BD Bioscience, clone EH12.1, BUV737, catalog no. 612792, 1:50 dilution); TOX (Miltenyi, clone REA473, PE, catalog no. 130-120-716, 1:50 dilution); EOMES (Invitrogen, clone WD1928, PE-eFluor610, catalog no. 61-4877-41, 1:50 dilution); TCF1/TCF7 (Cell Signaling, clone C63D9, Pacific Blue, catalog no. 9066 S, dilution 1:50); 4-1BB (BD Bioscience, clone 4B4-1, BV480, catalog no. 746700, dilution 1:50); TRAF1 (BD Bioscience, clone 1F3, AF647, catalog no. 566738, dilution 1:25); CD95 (BioLegend, clone DX2, APC/Fire 750, catalog no. 305638, dilution 1:50); CD39 (BD Bioscience, clone TU66, BUV661, catalog no. 749967, dilution1:50); CD3 (BioLegend, clone UCHT1, BV570, catalog no. 300436, dilution 1:25); CD4 (BioLegend, clone SK3, SparkBlue550, catalog no. 344656, dilution 1:400); CD8 (BD Bioscience, clone RPA-T8, BUV805, catalog no. 749366, dilution 1:100); TIM3 (BioLegend, clone F38-2E2, BV650, catalog no. 345028, dilution 1:100); Ki67 (BD Bioscience, clone B56, BV711, catalog no. 563755, dilution 1:200); PRF1 (BioLegend, clone B-D48, AF700, catalog no. 353324, dilution 1:100); and CD45RA (BD Bioscience, clone HI100, BVU395, catalog no. 740298, dilution 1:100).

### scATAC-seq quality control and filtering

scATAC-seq data from eight patients and three tissue types (PBMC, tumor and adjacent normal) underwent quality control analysis and filtering based on enrichment of ATAC-seq accessibility at TSSs and the number of unique fragments per cell, as described in ref. ^27^. TSS positions were acquired from the TxDb.Hsapiens.UCSC.hg38.knownGene Bioconductor package. Potential doublets were identified and removed using the software tool ArchR^28^, following the instructions in the manual for ArchR v.1.0.1.

### Genome-accessibility-based cell clustering

Two rounds of feature selection, dimension reduction and cell clustering were performed as previously described^27,61^. For immune cell clustering, first, a tiling window-by-cell counting matrix was constructed by counting the Tn5 insertion overlaps per window for each cell, using a tiling window size of 2.5 kb across the human genome (hg38). The matrix was then binarized and dimension reduction was conducted by computing the term frequency-inverse document frequency (TF-IDF) transformation. The resulted TF-IDF matrix then underwent irlba singular value decomposition (SVD) and the second to 25th dimensions were retained; cells were then clustered using Seurat’s SNN graph clustering (v.3.0) with a starting resolution of 0.8, requiring the minimum size of a cluster to be 200. In this specific case, a final resolution of 0.8 was used in the first round of clustering as the criterion for minimum cluster size was met. For the second round, peaks were called in each crude cell cluster obtained from the first round using MACS2^62^, and a union peak set was collected by combining peaks from all the cell clusters. A peak-by-cell counting matrix was then constructed by counting the Tn5 insertion overlaps per peak for each cell, and dimension reduction and cell clustering were performed as in round one. During this round of clustering, resolutions of 0.1, 0.2, 0.3, 0.4, 0.5, 0.6, 0.7, 0.8, 1.0 and 2.0 were tested, and a final resolution of 0.8 was chosen. For the subclustering of T cells, all the other parameters remained the same except that the minimum size of a cluster was set to be 250 for the first round of clustering, and a final resolution of 0.5 was used to meet this criterion.

### Gene activity score

Gene activity scores were calculated based on a model described previously^28^. ATAC-seq signals from the whole gene body were considered, and additional signals with bi-directional exponential decay weight from the gene TSS (extended 5 kb upstream) and the gene transcription termination site up to 100 kb were scaled and incorporated after filtering signals from overlapping neighbor gene boundaries.

### TF motif enrichment calculation

TF motif enrichment for peaks in each cell cluster was calculated using chromVAR^35^. CIS-BP motifs of 857 TFs were collected from chromVAR motifs ‘human_pwms_v2’, and the motif matches within peaks as well as raw signal counts for all peaks were used. GC bias-corrected deviation scores and variability for each TF motif were then calculated as instructed by chromVAR.

### Pseudotime analysis along dysfunction clusters

Cells from dysfunction clusters were ordered in pseudotime, and changes in gene score and TF motifs along the pseudotime trajectory were calculated and visualized in the form of heatmaps using the ArchR software tool^28^.

### scRNA-seq analysis

scRNA-seq data from four RCC patients and three tissue types were filtered using Seurat (v.3.0)^44^ according to the instructions with the following criteria: nFeature_RNA > 500 & nFeature_RNA < 5000 & percent.mito < 0.25 & nCount_RNA < 50000. Cells with multiple TCR alpha or beta chains were filtered as well. For T cell subclustering, T cells were recognized as having constructive TCR beta chains. Filtered data from four RCC patients were integrated and batch-effect-corrected, clustered, analyzed and visualized following the standard dataset integration and analysis workflow in Seurat v.3.0. Sets (modules) of highly correlated genes in the scRNA-seq data were calculated using WGCNA^63^ from the four-patient scRNA-seq Seurat object constructed as described above, with the following parameters: power = 10,corType = “bicor”, networkType = “signed”, minModuleSize = 10, reassignThreshold = 0, mergeCutHeight = 0.15, numericLabels = F, maxBlockSize=47000, pamRespectsDendro = FALSE.

### TCR-seq analysis

TCR-seq data were analyzed using scRepertoire (v.1.0.2)^64^. A TCR clonotype is defined as the combination of genes comprising the TCR and the nucleotide sequence of the CDR3 region (gene + nucleotide) for paired TCR alpha and beta chains. Differential gene expression analysis between T cell populations with different clonotypes (or clonotype features) was performed using Seurat 3.0 (ref. ^44^). Comparisons of the degree of clonal expansion (expa index), extent of tissue migration of TCR clonotype (migr index) and extent of state (cell cluster type) transitions of TCR clonotype (tran index) for each cell cluster were performed using STARTRAC as described previously^6^. Counts of cells per cluster assigned by specific frequency ranges were obtained using scRepertoire (v.1.0.2)^64^.

### NF-κB signature analysis in TCGA and CheckMate cohorts

Bulk RNA-seq data for the TCGA KIRC cohort were obtained through the Broad GDAC Firehose (https://gdac.broadinstitute.org/), and clinical data for TCGA KIRC were obtained from cBioPortal for Cancer Genomics (https://www.cbioportal.org/)^65,66^. Bulk RNA-seq data and clinical data for the CheckMate cohorts were obtained from ref. ^23^. For both cohorts, the expression value of each gene was converted into a *z*-score (centered at 0). The NF-κB signature score was calculated as the average of the *z*-score expression values for all five genes in the signature. The association between the high and low signature score groups (defined as scores higher or lower than the median signature score of each cohort, respectively) and the overall survival time was evaluated by Kaplan–Meier analysis. Two-sided log-rank test was used to detect significant differences in overall survival between the high and low signature groups. All survival analyses were performed using the R packages survival (v.3.2-10) and survminer (v.0.4.9). For the CheckMate cohort, pairwise comparisons of NF-κB signature gene expression levels between clinical response groups were evaluated using two-sided Wilcoxon rank-sum test. For the TCGA KIRC cohort, the overall differences in levels of NF-κB signature gene expression among clinical stages were evaluated using Kruskal–Wallis test, and for pairwise comparisons between different stages a two-sided Wilcoxon rank-sum test was performed. All tests were performed using the R environment (R v.4.0.3).

### Integration of paired four-patient scATAC-seq and scRNA-seq data

We used Seurat v.3.0 and Signac (v.1.5.0) to integrate scATAC-seq and scRNA-seq datasets collected from the same samples of four patients, according to the method introduced by Stuart and colleagues^44^. Integration was performed following the ‘Integrating scRNA-seq and scATAC-seq data vignette’ instructions in Seurat. Cells with prediction score greater than 0.5 were classified as high-confidence cells projected from scATAC-seq onto the UMAP derived from the scRNA-seq dataset. An alluvial plot showing the projection of cells from the scATAC-seq platform onto the UMAP derived from the scRNA-seq data was generated using the ggalluvial package (v.0.12.3) in R.

### Functional analysis of *cis*-regulatory regions

GREAT gene ontology (v.4.0.4) (http://great.stanford.edu/public/html/)^33^ was used to assign associated gene ontology terms to *cis*-regulatory regions that were most accessible (defined as FDR less than 0.01 and log_2_(fold change) greater than 1.5 compared with other clusters) in each T cell subcluster in the scATAC-seq data. The basal plus extension model was chosen to associate genes with *cis*-regulatory genomic regions. hg38 genome assembly was used in the analysis. Heatmaps comparing enrichment of GO terms across the clusters was generated using Morpheus (https://software.broadinstitute.org/GENE-E/).

### Statistics and reproducibility

No statistical method was used to predetermine sample sizes, but our sample sizes were similar to those reported in previous publications^22,23,25^. No data were excluded from the analyses. The experiments were not randomized. The investigators were not blinded to allocation during experiments or outcome assessment. Data collection and analysis were not performed blind to the conditions of the experiments. Statistical analysis was performed in R v.4.0.3. In general, comparisons of numerical variables between groups were carried out with a nonparametric approach (two-tailed Wilcoxon rank-sum test, Kruskal–Wallis test). Statistical analysis for flow cytometry and dysfunction score was performed by one-way analysis of variance (ANOVA). Data distribution was not formally tested to be normal. Gene ontology term enrichment was calculated using either the binomial test or the hypergeometric test. Survival curves were analyzed by the Kaplan–Meier method and compared using the log-rank test.

### Reporting summary

Further information on research design is available in the Nature Research Reporting Summary linked to this article.

## Supplementary information

Supplementary Information Supplementary Table 1

Reporting Summary

## Data Availability

Single-cell ATAC-seq, RNA-seq and TCR-seq data that support the findings of this study have been deposited in the Gene Expression Omnibus under accession code GSE181064. Bulk RNA-seq data for the TCGA KIRC cohort were obtained through the Broad GDAC Firehose (https://gdac.broadinstitute.org/), and clinical data for TCGA KIRC were obtained from cBioPortal for Cancer Genomics (https://www.cbioportal.org/)^65,66^. Bulk RNA-seq data and clinical data for the CheckMate cohorts were obtained from ref. ^23^. Source data are provided with this paper. All other data supporting the findings of this study are available from the corresponding author on reasonable request.

## Source data

Source Data Fig. 2 Statistical source data.
Source Data Fig. 3 Statistical source data.
Source Data Fig. 4 Statistical source data.
Source Data Fig. 5 Statistical source data.
Source Data Extended Data Fig. 1 Statistical source data.
Source Data Extended Data Fig. 2 Statistical source data.
Source Data Extended Data Fig. 4 Statistical source data.
Source Data Extended Data Fig. 5 Statistical source data.
Source Data Extended Data Fig. 6 Statistical source data.
Source Data Extended Data Fig. 7 Statistical source data.
Source Data Extended Data Fig. 8 Statistical source data.
Source Data Extended Data Fig. 10 Statistical source data.
