# Supplementary material for: A single-cell map of dynamic chromatin landscapes of immune cells in renal cell carcinoma
Source: Nat Cancer. 2022 Jun 6;3(7):885–98. doi: 10.1038/s43018-022-00391-0 (PMC9325682; doi:10.1038/s43018-022-00391-0)
Supplement: Supplementary file 1 — Supplementary Table 1 [file 43018_2022_391_MOESM1_ESM.pdf]

nature portfolio

https://doi.org/10.1038/s43018-022-00391-0

Supplementary information

A single-cell map of dynamic chromatin landscapes of immune cells in renal cell carcinoma

In the format provided by the authors and unedited

Source	Assay	Subject ID	Stage	Age	Sex	scATAC-seq				scRNA-seq			
						Total filtered CD45$^+$ cells	Tumor	PBMC	Normal adjacent tissue	Total filtered CD45$^+$ cells	Tumor	PBMC	Normal adjacent tissue
Avaden	Single-cell analysis	1002300	Tlb,N0,M0	79	F	6,854	3,179	2,011	1,664	27,843	10,010	9,109	8,724
Avaden	Single-cell analysis	2001055	Tlb,N0,M0	60	M	2,179	1,085	1,094	N/A	N/A	N/A	N/A	N/A
Avaden	Single-cell analysis	2001077	Tla,N0,M0	61	M	3,066	1,677	1,389	N/A	N/A	N/A	N/A	N/A
Avaden	Single-cell analysis	2001215	Tla,N0,M0	65	M	3,744	2,188	1,556	N/A	N/A	N/A	N/A	N/A
Avaden	Single-cell analysis	2001221	Tla,N0,M0	64	M	2,613	1,910	703	N/A	N/A	N/A	N/A	N/A
Avaden	Single-cell analysis	7001031	Tla,N0,M0	45	M	4,123	1,858	1,907	358	21,114	13,340	3,861	3,913
Avaden	Single-cell analysis, Flow cytometry	7001025	Tlb,N0,M0	45	M	5,084	1,892	1,739	1,453	34,215	9,839	15,708	8,668
Avaden	Single-cell analysis, Flow cytometry	1002310	Tlb,N0,M0	65	M	7,028	3,062	2,395	1,571	24,066	7,787	9,485	6,794
Discovery Life science	Flow cytometry	120891915	I	72	M								
Discovery Life science	Flow cytometry	110003259	I	52	M								
Discovery Life science	Flow cytometry	200003349	I	49	M								
Discovery Life science	Flow cytometry	121576700	I	66	M								
Discovery Life science	Flow cytometry	110042469	I	56	M								
Discovery Life science	Flow cytometry	200002022	I	76	F								
Discovery Life science	Flow cytometry	200001101	I	65	F								
Discovery Life science	Flow cytometry	110003572	I	68	F								
Discovery Life science	Flow cytometry	200000629	I	57	M								
Discovery Life science	Flow cytometry	110002687	I	52	M								
Discovery Life science	Flow cytometry	121222634	I	28	M								
Discovery Life science	Flow cytometry	200000912	I	47	M								
Discovery Life science	Flow cytometry	121441278	I	61	M								
Discovery Life science	Flow cytometry	110042455	I	62	M								

Supplementary Table 1. Patient information, sample source and cell numbers for the ccRCC samples used in this study.
