# Supplementary material for: A single-cell map of dynamic chromatin landscapes of immune cells in renal cell carcinoma
Source: Nat Cancer. 2022 Jun 6;3(7):885–98. doi: 10.1038/s43018-022-00391-0 (PMC9325682; doi:10.1038/s43018-022-00391-0)
Supplement: Supplementary file 2 — Reporting Summary [file 43018_2022_391_MOESM2_ESM.pdf]

nature portfolio

Corresponding author(s): Dimitris Skokos

Last updated by author(s): Mar 24, 2022

Reporting Summary

Nature Portfolio wishes to improve the reproducibility of the work that we publish. This form provides structure for consistency and transparency in reporting. For further information on Nature Portfolio policies, see our Editorial Policies and the Editorial Policy Checklist.

Statistics

For all statistical analyses, confirm that the following items are present in the figure legend, table legend, main text, or Methods section.

n/a	Confirmed	
☐	☒	The exact sample size (*n*) for each experimental group/condition, given as a discrete number and unit of measurement
☐	☒	A statement on whether measurements were taken from distinct samples or whether the same sample was measured repeatedly
☐	☒	The statistical test(s) used AND whether they are one- or two-sided<br>*Only common tests should be described solely by name; describe more complex techniques in the Methods section.*
☒	☐	A description of all covariates tested
☐	☒	A description of any assumptions or corrections, such as tests of normality and adjustment for multiple comparisons
☐	☒	A full description of the statistical parameters including central tendency (e.g. means) or other basic estimates (e.g. regression coefficient) AND variation (e.g. standard deviation) or associated estimates of uncertainty (e.g. confidence intervals)
☐	☒	For null hypothesis testing, the test statistic (e.g. *F*, *t*, *r*) with confidence intervals, effect sizes, degrees of freedom and *P* value noted<br>*Give P values as exact values whenever suitable.*
☒	☐	For Bayesian analysis, information on the choice of priors and Markov chain Monte Carlo settings
☒	☐	For hierarchical and complex designs, identification of the appropriate level for tests and full reporting of outcomes
☒	☐	Estimates of effect sizes (e.g. Cohen's *d*, Pearson's *r*), indicating how they were calculated

Our web collection on statistics for biologists contains articles on many of the points above.

Software and code

Policy information about availability of computer code

Data collection	No software was used for data collection
Data analysis	Cell Ranger v3.0.2<br>Seurat v3.0<br>Codes from Satpathy et al., Nature Biotechnology, 2019 (https://github.com/GreenleafLab/10x-scATAC-2019)<br>MACS2<br>ArchR v0.9.5<br>chromVAR 1.14.0<br>WGCNA v1.69<br>scRepertoire v1.0.2<br>STARTRAC v0.1.0<br>R 3.6.3 and R 4.0.3<br>GREAT GO 4.0.4<br>survival v3.2-10<br>survminer v0.4.9<br>Signac v1.5.0<br>ggalluvial package v0.12.3<br>Morpheus (https://software.broadinstitute.org/morpheus/)

For manuscripts utilizing custom algorithms or software that are central to the research but not yet described in published literature, software must be made available to editors and reviewers. We strongly encourage code deposition in a community repository (e.g. GitHub). See the Nature Portfolio guidelines for submitting code & software for further information.

Data

Policy information about availability of data

All manuscripts must include a data availability statement. This statement should provide the following information, where applicable:

- Accession codes, unique identifiers, or web links for publicly available datasets
- A description of any restrictions on data availability
- For clinical datasets or third party data, please ensure that the statement adheres to our policy

Single-cell ATAC-seq, RNA-seq, and TCR-seq data that support the findings of this study have been deposited in the Gene Expression Omnibus (GEO) under accession code GSE181064.
Bulk RNA-seq data for the TCGA ccRCC cohort (KIRC) was obtained through the Broad GDAC Firehose (https://gdac.broadinstitute.org/), and clinical data for TCGA KIRC was obtained from cBioPortal for Cancer Genomics (https://www.cbioportal.org/) (Cerami et al, Cancer Discov, 2012; Gao et al, Sci Signal, 2013). Bulk RNA-seq data and clinical data for the CheckMate cohorts were obtained from (Braun et al, Cancer Cell, 2021)
human GRCh38 genome assembly was used for read mapping.

Field-specific reporting

Please select the one below that is the best fit for your research. If you are not sure, read the appropriate sections before making your selection.

☒ Life sciences ☐ Behavioural & social sciences ☐ Ecological, evolutionary & environmental sciences

For a reference copy of the document with all sections, see nature.com/documents/nr-reporting-summary-flat.pdf

Life sciences study design

All studies must disclose on these points even when the disclosure is negative.

Sample size	No statistical method was used to pre-determine sample sizes but our sample sizes are similar to those reported in previous publications (Bi et al., Cancer Cell, 2021; Braun et al., Cancer Cell, 2021;Krishna et al, Cancer Cell, 2021)
Data exclusions	No data were excluded from the analyses
Replication	Due to the limited biological material available from patients, replications was no possible. However, for sequencing studies and flow cytometry, multiple independent patient samples were tested as indicated in text, figure legends and methods.
Randomization	As no therapeutic interventions were undertaken, randomization is not relevant.
Blinding	The methods employed in this study involve unbiased profiling of the epigenome/transcriptome, and flow cytometry. There was no therapeutic intervention or expected outcome prior to the corresponding analysis, therefore blinding is not relevant

Reporting for specific materials, systems and methods

We require information from authors about some types of materials, experimental systems and methods used in many studies. Here, indicate whether each material, system or method listed is relevant to your study. If you are not sure if a list item applies to your research, read the appropriate section before selecting a response.

Materials & experimental systems

n/a	Involved in the study
☐	☒ Antibodies
☒	☐ Eukaryotic cell lines
☒	☐ Palaeontology and archaeology
☒	☐ Animals and other organisms
☐	☒ Human research participants
☒	☐ Clinical data
☒	☐ Dual use research of concern

Methods

n/a	Involved in the study
☒	☐ ChIP-seq
☐	☒ Flow cytometry
☒	☐ MRI-based neuroimaging

Antibodies

Antibodies used	Listed as - Antibody: Supplier, Clone name, Fluorochrome, Catalogue number, Dilution<br>Flow cytometry antibodies:<br>PD-1: BD Biosciences, EH12.1, BUV737, 612792, 1:50<br>TOX: Miltenyi, REA473, PE, 130-120-716, 1:50

EOMES: Invitrogen, WD1928, PE-eFluor610, 61-4877-42, 1:50
CD39: BD Bioscience, TU66, BUV661, 749967, 1:50
TIM3: BioLegend, F38-2E2, BV650, 345028, 1:100
4-1BB: BD Bioscience, 4B4-1, BV480, 746700, 1:50
CD95 (FAS): BioLegend, DX2, APC/Fire750, 305638, 1:50
TRAF1: BD Bioscience, 1F3, eFluor660, 566738, 1:25
PRF1: BioLegend, B-D48, AF700, 353324, 1:100
TCF7: Cell Signaling, C63D9, Pacific Blue, 9066S, 1:50
CD45RA: BD Bioscience, HI100, BUV395, 740298, 1:100
CD4: BioLegend, SK3, SparkBlue 550, 344656, 1:400
CD3: BioLegend, UCHT1, BV570, 300436, 1:25
CD8: BD Bioscience, RPA-T8, BUV805, 749366, 1:100
Ki67:BD Bioscience, B56, BV711, 563755, 1:200
FAM-VAD-FMK poly caspases: Invitrogen, V35117

Validation

All the antibodies used in this study were commercial antibodies, with validation procedures from the manufacturer described in the following sites: https://www.biolegend.com; https://www.bdbiosciences.com/en-us; https://www.thermofisher.com/us/en/home/brands/invitrogen.html; https://www.cellsignal.com; https://www.miltenyibiotec.com/US-en/#gref

Human research participants

Policy information about studies involving human research participants

Population characteristics

All patients had Stage I renal cell carcinoma with clear cell histology. Information about the age and sex of the patients is provided in Supplementary Table 1. None of the patients received therapy prior to tumor resection

Recruitment

There is no evidence that this study was prone to self-selection bias

Ethics oversight

Consented patient samples were procured from commercial vendors (Avaden BioSciences and Discovery Life Sciences)

Note that full information on the approval of the study protocol must also be provided in the manuscript.

Flow Cytometry

Plots

Confirm that:

☒ The axis labels state the marker and fluorochrome used (e.g. CD4-FITC).

☒ The axis scales are clearly visible. Include numbers along axes only for bottom left plot of group (a 'group' is an analysis of identical markers).

☒ All plots are contour plots with outliers or pseudocolor plots.

☒ A numerical value for number of cells or percentage (with statistics) is provided.

Methodology

Sample preparation

Consented patient samples were procured from commercial vendors (Avaden BioSciences and Discovery Life Sciences). The sample processing protocol is described in Methods

Instrument

BD FACSymphony A5, Cytek Aurora

Software

OMIQ

Cell population abundance

The abundance of CD45+ determined by testing the sorted cells with FACS, reached >99%

Gating strategy

Gating strategy used to derive data in Fig. 4e. Viable cells were gated for CD45+ cells, T cells (CD2+CD3+), CD3+CD8+CD4- cells, and the % cells expressing PD-1, TOX, 4-1BB, FAS, TRAF1 and combinations was assessed. Positive and negative expression was based on isotype and FMO controls.

☒ Tick this box to confirm that a figure exemplifying the gating strategy is provided in the Supplementary Information.
